# *Plasmodium vivax*-like genome sequences shed new insights into *Plasmodium vivax* biology and evolution

**DOI:** 10.1371/journal.pbio.2006035

**Published:** 2018-08-24

**Authors:** Aude Gilabert, Thomas D. Otto, Gavin G. Rutledge, Blaise Franzon, Benjamin Ollomo, Céline Arnathau, Patrick Durand, Nancy D. Moukodoum, Alain-Prince Okouga, Barthélémy Ngoubangoye, Boris Makanga, Larson Boundenga, Christophe Paupy, François Renaud, Franck Prugnolle, Virginie Rougeron

**Affiliations:** 1 MIVEGEC, IRD, CNRS, University of Montpellier, Montpellier, France; 2 Wellcome Trust Sanger Institute, Wellcome Trust Genome Campus, Cambridge, United Kingdom; 3 Institute of Infection, Immunity and Inflammation, University of Glasgow, College of Medical, Veterinary and Life Sciences, Glasgow, United Kingdom; 4 Centre International de Recherches Médicales de Franceville, Franceville, Gabon; Pennsylvania State University, United States of America

## Abstract

Although *Plasmodium vivax* is responsible for the majority of malaria infections outside Africa, little is known about its evolution and pathway to humans. Its closest genetic relative, *P*. *vivax*-like, was discovered in African great apes and is hypothesized to have given rise to *P*. *vivax* in humans. To unravel the evolutionary history and adaptation of *P*. *vivax* to different host environments, we generated using long- and short-read sequence technologies 2 new *P*. *vivax*-like reference genomes and 9 additional *P*. *vivax*-like genotypes. Analyses show that the genomes of *P*. *vivax* and *P*. *vivax*-like are highly similar and colinear within the core regions. Phylogenetic analyses clearly show that *P*. *vivax*-like parasites form a genetically distinct clade from *P*. *vivax*. Concerning the relative divergence dating, we show that the evolution of *P*. *vivax* in humans did not occur at the same time as the other agents of human malaria, thus suggesting that the transfer of *Plasmodium* parasites to humans happened several times independently over the history of the *Homo* genus. We further identify several key genes that exhibit signatures of positive selection exclusively in the human *P*. *vivax* parasites. Two of these genes have been identified to also be under positive selection in the other main human malaria agent, *P*. *falciparum*, thus suggesting their key role in the evolution of the ability of these parasites to infect humans or their anthropophilic vectors. Finally, we demonstrate that some gene families important for red blood cell (RBC) invasion (a key step of the life cycle of these parasites) have undergone lineage-specific evolution in the human parasite (e.g., reticulocyte-binding proteins [RBPs]).

## Author summary

Among the 5 species responsible for malaria in humans, *Plasmodium vivax* is the most prevalent outside Africa. It causes severe and incapacitating clinical symptoms with significant effects on human health. Yet little is known about its evolution, adaptation, and emergence in humans. The recent discovery in African great apes of its closest known relative—*P*. *vivax*-like—may help resolve the evolutionary history of *P*. *vivax*. This study aims to characterize the genome of *P*. *vivax*-like isolated from infections of the closest ape relative to humans to get a better understanding of the evolution of this parasite. A total of 11 *P*. *vivax*-like samples were obtained from infected chimpanzee blood samples and an infected mosquito collected in Gabon. We generated, and here present, the first 2 new genomes of *P*. *vivax*-like and 9 additional draft sequences. Genome-wide analyses provide new insights into the biology and adaptive evolution of *P*. *vivax* to different host species. Indeed, they highlight lineage-specific evolution of some gene families involved in key steps of the life cycle of *P*. *vivax*. Analyses also revealed that the divergence between *P*. *vivax* and *P*. *vivax*-like occurred before the one between *P*. *falciparum* and its sister species *P*. *praefalciparum*.

*Plasmodium vivax* is responsible for the majority of malaria infections in humans outside sub-Saharan Africa [1]. Traditionally, *P*. *vivax* has been neglected because it causes lower mortality in comparison with *P*. *falciparum* [2, 3]. Its ability to produce a dormant liver-stage form (hypnozoite), responsible for relapsing infections, makes it a challenging public health issue for malaria elimination. The recent emergence of antimalarial drug resistance [4] as well as the discovery of severe and even fatal human cases [2,5,6] has renewed interest in this enigmatic species, including its evolutionary history and its origin in humans.

Earlier studies placed the origin of *P*. *vivax* in humans in Southeast Asia (“Out of Asia” hypothesis) based on its phylogenetic position in a clade of parasites infecting Asian monkeys [7]. At that time, the closest known relative of *P*. *vivax* was considered to be *P*. *cynomolgi*, an Asian monkey parasite [8]. However, this hypothesis was recently challenged with the discovery of another *Plasmodium* species, genetically closer to *P*. *vivax* than *P*. *cynomolgi*, circulating in African great apes (chimpanzees and gorillas) [9,10]. This new lineage (hereafter referred to as *P*. *vivax*-like) was considered to have given rise to *P*. *vivax* in humans following the transfer of parasites from African apes [10]. But this “Out of Africa” hypothesis is still debated. Moreover, a spillover of *P*. *vivax*-like parasites to humans has been recently documented, thus making possible the release of new strains in new host species, specifically in human populations [9].

In this context, it seemed fundamental to characterize the genome of the closest ape relative to the human *P*. *vivax* parasite in order to get a better understanding of the evolution of this parasite and also to identify the key genetic changes explaining the emergence of *P*. *vivax* in human populations.

## Genome assemblies

Eleven *P*. *vivax*-like genotypes were obtained from 2 different kinds of samples: 10 infected chimpanzee blood samples collected during successive routine sanitary controls of chimpanzees living in the Park of La Lékédi (a sanctuary in Gabon) and 1 infected *Anopheles* mosquito (*An*. *moucheti*) collected during an entomological survey carried out in the same park (S1 Table) [11]. For blood samples, white blood cells were depleted using the CF11 method [12] to reduce the amount of host DNA. After DNA extraction, samples were subjected to whole-genome amplification (WGA) in order to obtain sufficient parasite DNA for library preparation. Sequencing was then performed using short-read Illumina technology. For one sample (Pvl06), long-read sequencing (Pacific Biosciences [PacBio] technology) was performed in order to get a better coverage of regions containing subtelomeric gene families.

Among the 11 samples, 10 presented mixed infections with other *Plasmodium* species (S1 and S2 Tables). Four samples containing *P*. *gaboni* or *P*. *malariae*-like co-infections were used in other studies (see S1 Table) [13,14]. In order to obtain the *P*. *vivax*-like genotypes and to preclude errors due to co-infections with other *Plasmodium* species, sequencing reads were extracted based on their similarity to the reference genome sequence of *P*. *vivax*, PvP01 [15], and any reads mapping against a reference genome of a *Laverania* chimpanzee-infecting species (e.g., PrG01, PbilcG01, and PGAB01; see Otto and colleagues [13]) were removed (S2 Table). Concerning multiple infections with several *P*. *vivax*-like strains, only 2 (Pvl09 and Pvl10) seemed to be multiply infected as suggested by the reference allele frequency (RAF) distributions (see Materials and methods section and S1 Fig). In order to avoid any bias in the genomic analysis due to multiple infections with several strains of *P*. *vivax*-like, only 1 variant was extracted per sample. This was done by considering the allele with the highest frequency in the calling and filtering analysis of Single Nucleotide Variants (SNVs) (see Materials and methods section). Sequencing reads from 2 samples, one obtained using Illumina sequencing, Pvl01, and another using PacBio technology, Pvl06, were used to perform de novo genome assemblies and were annotated to produce reference genomes for *P*. *vivax*-like (S1 Table). Of the 2 assemblies, Pvl01 is of considerably higher quality (4,570 one-to-one orthologues to the PvP01 reference genome compared with 2,925 for Pvl06 [Table 1]). Both assemblies consist of 14 supercontigs (corresponding to the 14 *P*. *vivax* chromosomes)—and 1,176 and 351 unassigned contigs—comprising a total of 27.5 Mb and 18.8 Mb in size, respectively, for Pvl01 and Pvl06, respectively. After annotation with Companion [16], these 2 genomes contained 5,532 and 4,953 annotated genes (Table 1). We obtained for the 9 remaining samples between 2.9 and 86 million of reads that paired with the *P*. *vivax* PvP01 reference genome (mean ± SD = 2.17 ± 25.71), with a mean depth per high-quality position ranging from 13.98 to 335.1 (mean ± SD = 93.43 ± 99.51; see S3 Table). These genome sequences were used for SNV calling for population genetic and phylogenetic analyses.

**Table 1:** Genome features of the *P*. *vivax*-like Pvl01 (Illumina HiSeq sequenced) and Pvl06 (PacBio sequenced) strains, *P*. *vivax* reference strains SalI and PvP01 [15], *P*. *cynomolgi* B and M isolates [8,17], and *P*. *knowlesi* H strain [18].

	*P*. *vivax*-like (Pvl01)	*P*. *vivax*-like (Pvl06)	*P*. *vivax* (PvP01)	*P*. *vivax* (SalI)	*P*. *cynomolgi* (B strain)	*P*. *cynomolgi* (M strain)	*P*. *knowlesi* (H strain)
**Assembly size (Mb)**	27.5	18.8	29	26.8	26.2	30.6	24.1
**Scaffolds**	14 (1,176)*	14 (351)*	14	14	14	14	14
**Overall GC content (%)**	44.9	45.8	39.8	42.3	40.3	37.3	37.5
**Number of genes**	5,532	4,953	6,642	6,690	5,722	6,632	5,188
**Gene density (gene/Mb)**	201.2	264.5	229	249.6	218.4	216.7	215.3
**Coverage**^#^	12.17×	34×	-	-	-	-	-
**One-to-one orthologues with PvP01**	4,570	2,925	-	5,178	4,870	5,222	4,804

*Unassigned contigs indicated in parentheses.

^#^Calculated as (L × *N*) ÷ G, where L is the read length (100 bp were considered for the Illumina sample, Pvl01), *N* the number of mapped reads, and G the size of the assembly.

**Abbreviations:** GC, guanine–cytosine; PacBio, Pacific Biosciences.

## Gene synteny and gene composition

Comparing the *P*. *vivax*-like reference genomes to those of *P*. *vivax* (PvP01 and SalI) [3,15], *P*. *cynomolgi* (B and M strains) [8,17], and *P*. *knowlesi* (H strain) [18] reveals several similarities, including a similar guanine–cytosine (GC) content and extensive collinearity and conservation of gene content and organization (Table 1). The *P*. *vivax*-like core genome sequences are completely syntenic to the *P*. *vivax* PvP01 reference genome sequence (S2 and S3 Figs).

Because multigene families are known to evolve extremely rapidly in their genome structure, obtaining the full genomes of species closer to human *P*. *vivax* is fundamental for a better understanding of its evolution, adaptation, and emergence in different host species. For *Plasmodium* parasites, most species-specific genes are part of large gene families, such as *var* genes in *P*. *falciparum* or *pir* genes that are present in all *Plasmodium* genomes studied [19,20]. Table 2 provides a summarized view of gene content and copy number of the main multigene families in *P*. *vivax*-like in comparison with *P*. *vivax*, *P*. *knowlesi*, and *P*. *cynomolgi*. Even if certain subtelomeric regions of our reference genomes (S2 and S3 Figs) are not complete, at least one copy of each major gene family was detected (Table 2). In comparison with *P*. *vivax* (and expected because of the partial subtelomeric sequencing coverage), the number of copies in each family was generally lower or equal in *P*. *vivax*-like. For these families, all genes were functional except for the Cytoadherence-linked asexual gene (*clag*) family. For the *clag* family, all genes are functional except the one situated on chromosome 8 for *P*. *vivax*-like (confirmed for both Pvl01 and Pvl06) (S4 Fig). The *clag* family, strictly conserved in malaria parasites, is an essential gene family in host–parasite interactions, playing a role in merozoite invasion, parasitophorous vacuole formation, and in the uptake of ions and nutrients from the host plasma [21,22]. The pseudogenization of the *clag* gene on chromosome 8 for *P*. *vivax*-like suggests that this species lost the *clag* gene during its adaptation to the ape host.

**Table 2:** Number of detected copies of multigene family members in the genomes of *P*. *vivax*-like (Pvl01 and Pvl06), *P*. *vivax* strains SalI and PvP01 [15], *P*. *cynomolgi* B and M isolates [8,17], and *P*. *knowlesi* H strain [18].

	*P*. *vivax*-like (Pvl01)*	*P*. *vivax*-like (Pvl06)*	*P*. *vivax* (PvP01)	*P*. *vivax* (SalI)	*P*. *cynomolgi* (B strain)	*P*. *cynomolgi* (M strain)	*P*. *knowlesi* (H strain)
***vir*/*pir* (subtelomeric)**	148	14	1,212	303	265	1,373	64
***msp3* (central)**	9	0	12	11	12	14	3
***msp7* (central)**	11	12	13	13	13	11	5
***dbp* (subtelomeric)**	1	0	2	1	2	2	3
***rbp* (subtelomeric)**	9	3	10	9	8	6	2
***Pv-fam-a (trag)* (subtelomeric)**	34	36	40	34	36	39	29
***Pv-fam-e (rad)* (subtelomeric)**	38	15	40	34	27	27	16
***pst-A* (subtelomeric and central)**	6	3	10	11	9	8	7
***etramp* (subtelomeric)**	7	4	9	10	9	9	9
***clag* (RhopH-1) (subtelomeric)**	3	2	3	3	2	2	2
***PvSTP1* (subtelomeric)**	4	0	10	9	3	51	0
***Phist* (*Pf-fam-b*) (subtelomeric)**	20	12	84	64	48	54	15
***sera* (central)**	13	7	13	13	13	13	7

*For *P*. *vivax*-like Pvl01 and Pvl06, a nonexhaustive list of family genes is represented because only partial genomes were obtained. Pseudogenized genes are included.

**Abbreviations:** CLAG, Cytoadherence-linked asexual gene; DBP, Duffy-binding protein; *etramp*, early transcribed membrane protein; *msp*, merozoite surface protein; Phist, *Plasmodium* helical interspersed subtelomeric; *pir*, *Plasmodium* interspersed repeat; *rbp*, reticulocyte-binding protein; *sera*, serine-repeat antigen; *STP1*, subtelomeric protein 1; *trag*, tryp-rich antigen.

During the life cycle of *Plasmodium* parasites, host RBC invasion is mediated by specific interactions between parasite ligands and host erythrocyte receptors. Two major multigene families are involved in RBC invasion: the Duffy-binding proteins (*dbp*) and the reticulocyte-binding proteins (*rbp*) multigene families [23]. DBP is a protein secreted by the micronemes of the merozoite stage that binds to the Duffy Antigen Receptor for Chemokine (DARC) to invade RBCs. *P*. *vivax* is characterized in its genome by 2 *dbp* genes (*dbp1* on chromosome 6 and *dbp2* on chromosome 1) that seem to be essential for RBC invasion, as demonstrated by their inability to infect individuals not expressing the Duffy receptor on the surface of their RBCs (i.e., Duffy-negative individuals) [24–26]. In the reference genomes of *P*. *vivax*-like (Pvl01 and Pvl06), we observe the *dbp1* gene as in the *P*. *vivax* genome PvP01 (Table 2) and also in the other *Plasmodium* species (*P*. *knowlesi* and *P*. *cynomolgi*); however, we did not observe the *dbp2* gene (no read obtained mapping to this region). This observation was confirmed in the other genotypes sequenced in this study. Knowing that gorillas and chimpanzees are all described as Duffy positive [10], we propose that *P*. *vivax*-like parasites infect only Duffy-positive hosts, which could be associated with the absence of the *dbp2* gene. This would be in accordance with the fact that the only described transfer of *P*. *vivax*-like to humans was in a Caucasian Duffy-positive individual [9] and that no transfers of *P*. *vivax*-like have been recorded in Central African Duffy-negative populations despite the fact that they live in close proximity to infected ape populations [27].

**Fig 1:**
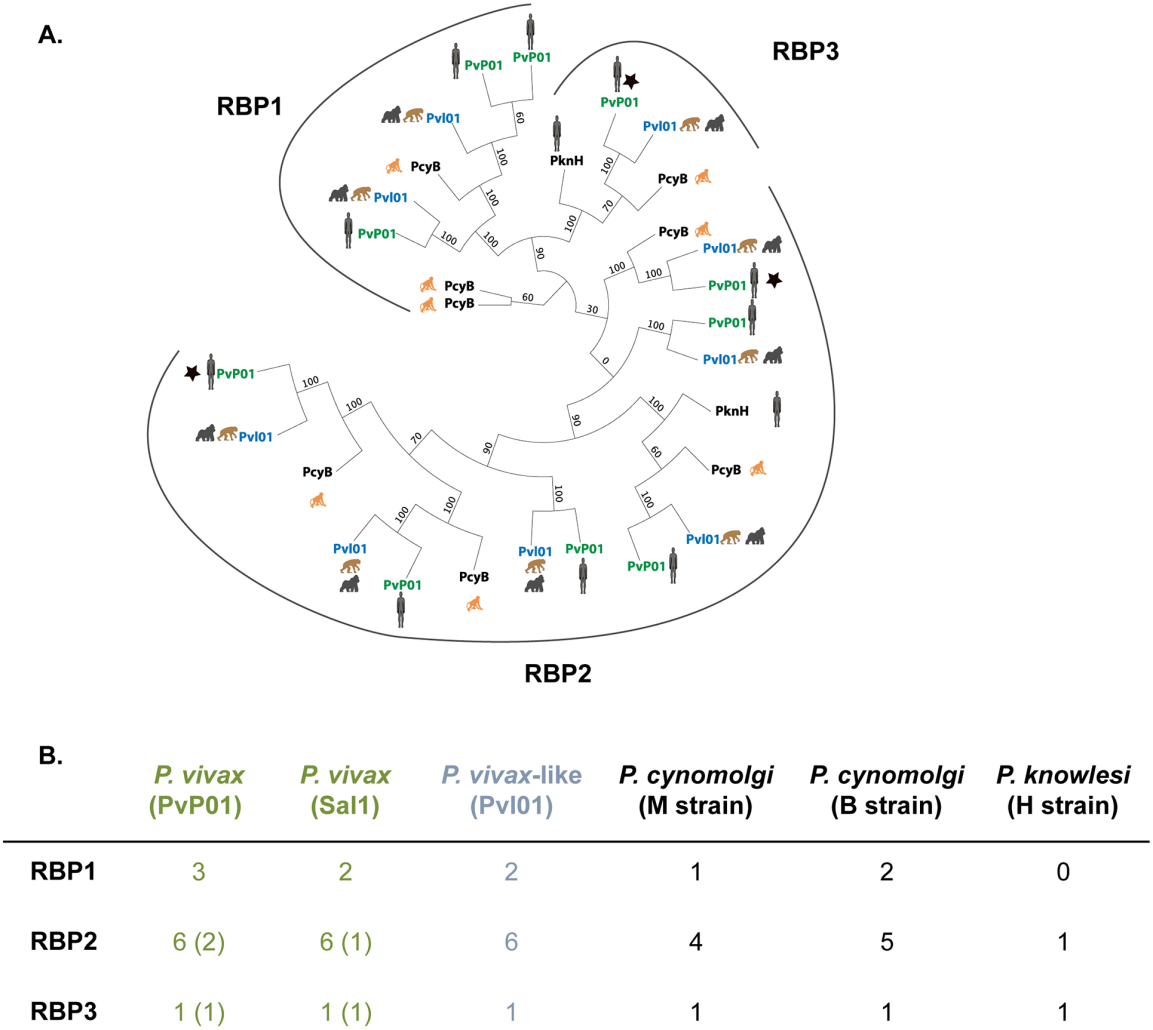
*rbp* genes in *P*. *vivax*-like and *P*. *vivax*. (A) Maximum likelihood phylogenetic tree of all full-length *rbp* genes in *P*. *vivax*-like Pvl01 (in blue), *P*. *vivax* SalI and PvP01 strains (in green), *P*. *cynomolgi* B strain, and *P*. *knowlesi* H strain (in black). Bootstrap values, calculated by RAxML bootstrapping posterior probability, are indicated. The different subclasses of *rbp* are indicated as *rbp1*, *rbp2*, and *rbp3*. The black stars indicate pseudogenes. The animal pictograms indicate the primate host. (B) Table representing the number of variants (including the ones that are pseudogenized) observed in each *rbp* subclass in *P*. *vivax*-like (Pvl01), *P*. *vivax* (SalI and PvP01), *P*. *cynomolgi* (B and M strains), and *P*. *knowlesi* (H strain). Pseudogenes detected among each subclass of *rbp* are indicated within each subclass between brackets. The alignment of the *rbp* sequences with their accession numbers indicated and the inferred RAxML tree are available as the supplemental files in S2 Data. RBP, reticulocyte-binding protein.

*rbp* genes encode a merozoite surface protein family present across all *Plasmodium* species and known to be involved in RBC invasion and host specificity [23]. Among *rbp* family genes, 3 gene classes (*rbp1*, *rbp2*, and *rbp3*) exist and are associated with the ability of *Plasmodium* parasites to invade different maturation stages of RBCs. In this study, comparison of the organization and characteristics of the *rbp* gene family between *P*. *vivax*, *P*. *vivax*-like, *P*. *knowlesi*, and *P*. *cynomolgi* (Fig 1 and Table 2) first reveals that gene classes RBP2 and RBP3 are ancestral to the divergence of all these species except *P*. *knowlesi*. Second, an expansion of the *rbp2* class is observed in the *P*. *vivax*/*P*. *vivax*-like*/P*. *cynomolgi* lineage (Fig 1A), suggesting that, in this lineage, specific expansion likely occurred during the evolution of these species. Finally, *rbp3* genes, which are supposed to confer the ability to infect normocytes, are functional in all species except in *P*. *vivax* (for which the gene is pseudogenized in both SalI and PvP01 strains), suggesting that *P*. *vivax* lost the ability to infect normocytes or has developed an ability to infect specifically only reticulocytes during its adaptation to human RBCs (Fig 1A and 1B).

## Phylogenetic relationships to other *Plasmodium* species and divergence time

**Fig 2:**
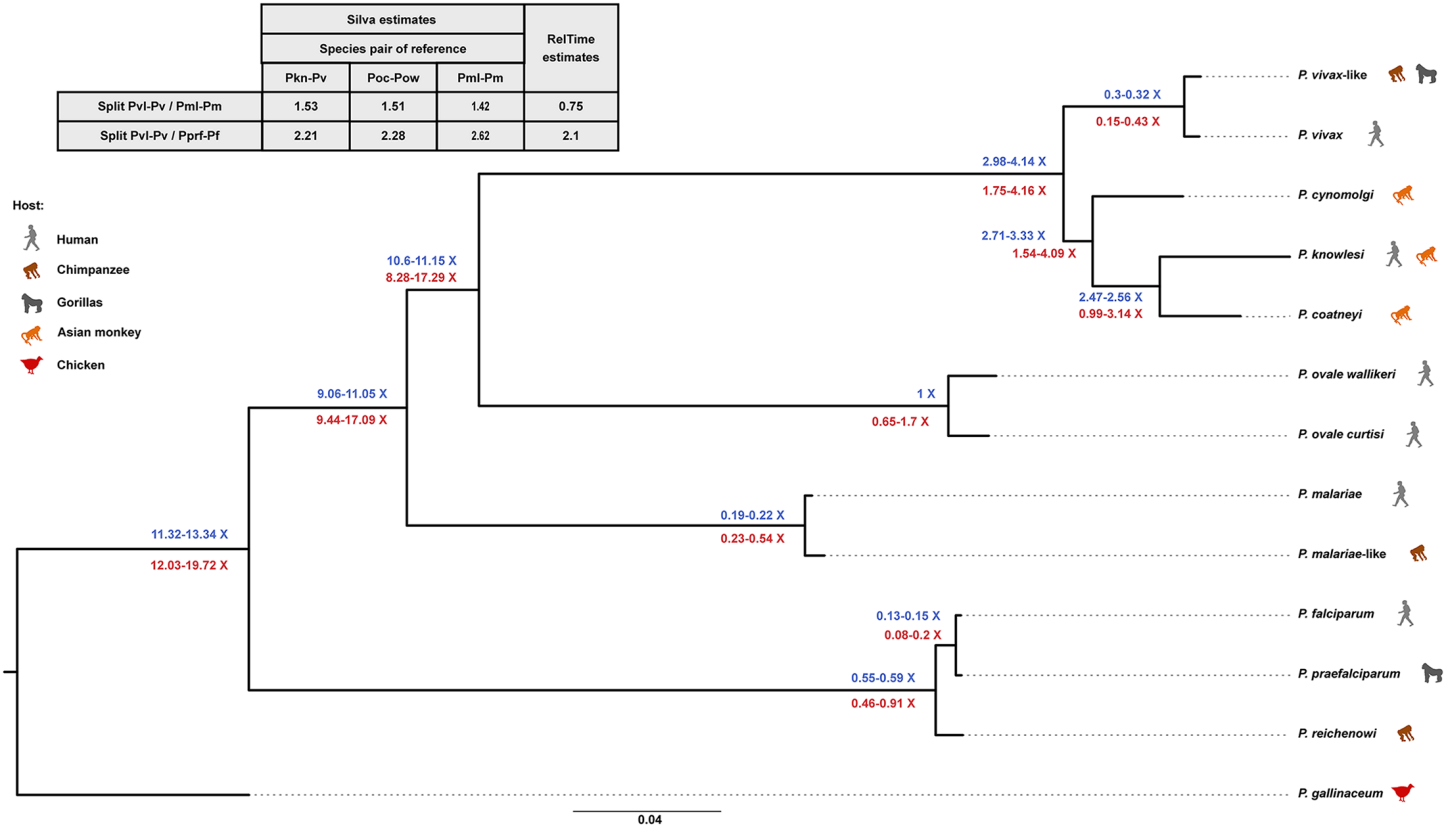
Relative divergence dating between *P*. *vivax* and *P*. *vivax*-like. Maximum likelihood phylogenetic tree of 13 *Plasmodium* species, including *P*. *vivax* and *P*. *vivax*-like. The analysis was based on an alignment of 2,784 one-to-one orthologous groups across the 13 *Plasmodium* reference genomes (see Materials and methods section). Values indicated at the nodes are the 95% CIs of the relative splits estimated using the method developed by Silva and colleagues [29] (values in blue) and the RelTime method [69] (values in red; ×100). Results are given for the analyses performed considering the JTT model of evolution. For the Silva method [29], we gave for the internal nodes the minimal and maximal values of the lower and upper limits of the 95% CI of all possible pairwise species combinations. The table gives the ratio between the relative divergence times of the human–nonhuman *Plasmodium* species pairs. The final alignment of the 2,784 one-to-one orthologues—excluding ambiguities, lox-complexity regions, and poorly aligned regions—and the tree are available as supplemental files in S3 Data. CI, confidence interval; JTT, Jones, Taylor, and Thornton; Pf, *P*. *falciparum*; Pkn, *P*. *knowlesi*; Pm, *P*. *malariae*; Pml, *P*. *malariae*-like; Poc, *P*. *ovale curtisi*; Pow, *P*. *ovale wallikeri*; Pprf, *P*. *praefalciparum*; Pv, *P*. *vivax*; Pvl, *P*. *vivax*-like.

Conservation of the gene content between *P*. *vivax*-like with the other primate-infective *Plasmodium* species has enabled us to reconstruct with confidence the relationships between the different species and to estimate the relative age of the different speciation events. This analysis confirmed the position of *P*. *vivax*-like as the closest sister lineage of *P*. *vivax* (Fig 2).

Regarding the estimation of divergence times using genomic information, different methods were recently used for *Plasmodium*, such as the one implemented in Generalized Phylogenetic Coalescent Sampler (G-PhoCS) [28] or the one developed by Silva and colleagues [29]. G-PhoCS uses a Bayesian Markov Chain Monte Carlo (MCMC) approach to infer, based on the information provided by multiple loci, the divergence time between species. This method has been applied in 2 recent studies for *Plasmodium* parasites—one aiming at estimating the relative split times between the 2 *P*. *ovale* subspecies and between *P*. *malariae* and *P*. *malariae*-like [14], the other to estimate the divergence time within the *Laverania* subgenus, a subgenus including *P*. *falciparum* and all its closest ape relatives [13]. The Silva method is based on the estimate of the sequence divergence in different proteins and comparison of this divergence measured between different lineages [29]. In this method, the regression slope of the divergence between the proteins in 2 lineages reflects their relative age. The advantage is that it does not rely on an estimate of mutation rate. Finally, it has already been used in a recent study estimating avian and primate *Plasmodium* species divergence times [30]. Here, without calibration points and a good estimation of the *P*. *vivax* and other *Plasmodium* species substitution rates, our aim was to evaluate the divergence time between *P*. *vivax* and *P*. *vivax*-like relative to the divergence time between the other primate–nonprimate *Plasmodium* species pairs. To evaluate the influence of the approach on the estimations, we used 2 strategies to estimate divergence time between *P*. *vivax* and *P*. *vivax*-like relative to the other divergence events within the tree: the Silva method [29] and the RelTime method (see Materials and methods section for a description of the two methods) [31,32]. We first used the Silva method [29], checking for the influence of the model of evolution and the reference species pair on the analysis (see Materials and methods section). Because neither influenced the results of the relative ages of species pairs (see Fig 2, S5 Fig and S4 Table), we only report the results for the Silva method using the JTT (Jones, Taylor, and Thornton) model of evolution and the *P*. *ovale curtisi*–*P*. *ovale wallikeri* pair as the species pair of reference as in Böhme and colleagues in Fig 2 [30]. While the 2 methods are mostly in agreement (with RelTime showing larger confidence intervals [CIs]), they show a discrepancy concerning the relative divergence time of the *P*. *malariae* and *P*. *malariae*-like parasites (see Fig 2 and S4 Table). However, both methods suggest that the evolution of *P*. *vivax* in humans did not occur at the same time as the other human malaria agents of the *Plasmodium* genus (i.e., *P*. *malariae* and *P*. *falciparum*). The analyses show that the time of the split between *P*. *vivax* and *P*. *vivax*-like happened before the divergence between *P*. *falciparum* and *P*. *praefalciparum* (about 2.3 times earlier). It is unclear from our analyses whether the divergence between the 2 *P*. *malariae* species occurred before or after the divergence of the other 2 human–nonhuman parasite pairs. Unlike us, a previous study estimated, using the G-PhoCS method, the relative dating of the split between *P*. *reichenowi* (a chimpanzee parasite) and *P*. *falciparum* (*P*. *praefalciparum* was not available at that time) and that of the split between *P*. *malariae* and *P*. *malariae*-like (note that similar estimates were recently obtained using another set of data using the Silva method) [14] to have occurred at the same time. This discrepancy between methods suggests that the same strict molecular clock (which is a hypothesis of the Silva method) may not apply over the entire tree (especially for the *Laverania* subgenus because of their extremely low GC content in comparison with other *Plasmodium* species). Whatever the method used, all these estimates nevertheless suggest that the evolution of *P*. *vivax* in humans did not occur at the same time as the other human malaria species and that the transfer of *Plasmodium* parasites to humans may have happened several times independently over the history of the *Homo* genus [33–37].

## Relationships to worldwide human *P*. *vivax* isolates

**Fig 3:**
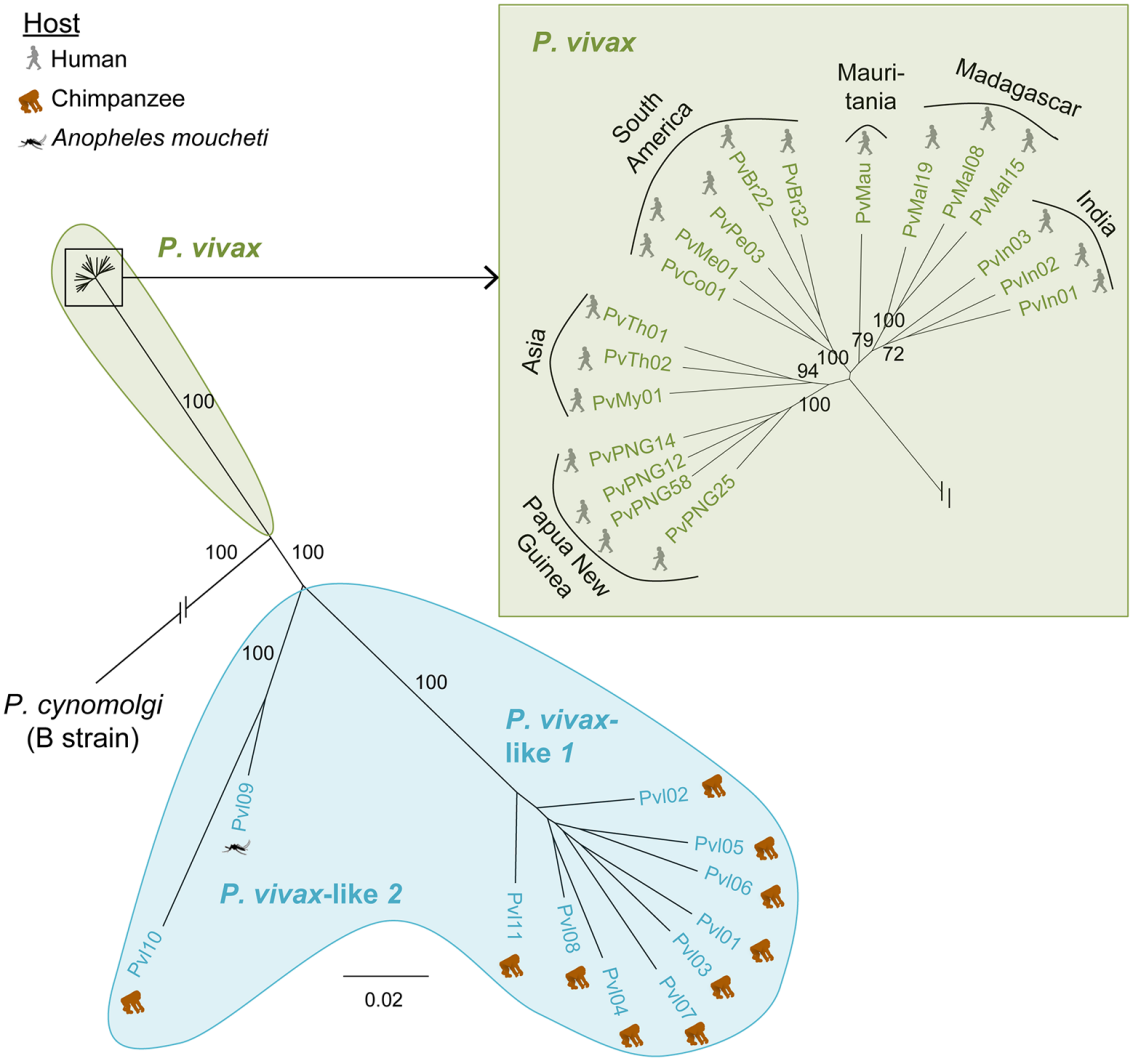
ML phylogenetic tree with 1,000 bootstraps computed by alignment to the *P*. *cynomolgi* B strain genome, based on 100,616 SNVs shared by 11 *P*. *vivax*-like and 19 *P*. *vivax* samples. A position was considered an SNV if at least one sample carried a different nucleotide compared with the PvP01 reference. No missing data were allowed, and a minimum depth of 5 reads per position was considered. To overcome issues relating to multiple infections, we considered the dominant infection only by selecting the dominant allele (see Materials and methods for details). Bootstrap values superior to 70% are indicated. The host in which the *Plasmodium* parasite was detected is indicated by the pictograms (human, chimpanzee, and *An*. *moucheti*). This phylogeny showed the presence of a significantly distinct clade (high bootstrap values associated with each clade) composed of *P*. *vivax*-like strains on one side (light blue) and human *P*. *vivax* isolates on the other side (light green). Data (the alignment and the tree file obtained by the ML phylogenetic analysis) can be found in S5 Data. ML, maximum likelihood; SNV, single nucleotide variant.

To analyze the relationship between our 11 *P*. *vivax*-like isolates and human *P*. *vivax*, we completed our dataset with 19 published human *P*. *vivax* genomes (S1 Table) [38]. All sequencing reads were aligned against the PvP01 reference genome [15], and SNVs were called and filtered as described in the Materials and methods section. Maximum likelihood phylogenetic trees were then produced based on 100,616 SNVs. Our results clearly demonstrate the presence of 2 significantly distinct genetic clades (with a bootstrap value of 100) composed of *P*. *vivax*-like strains on one side and human *P*. *vivax* isolates on the other side (Fig 3). This result differs from previous results suggesting that human strains formed a monophyletic clade within the radiation of ape *P*. *vivax*-like parasites [10].

One explanation for this difference with previous published results could be that it is due to a phenomenon called Incomplete Lineage Sorting (ILS) or to a lack of phylogenetic signal for phylogenies performed on a single or few genes. ILS is the discordance observed between some gene trees and the species or population tree due to the coalescence of gene copies in an ancestral species or population [39]. Such a phenomenon is often observed when species or population divergence is recent, which is the case for *P*. *vivax*/*P*. *vivax*-like [40,41]. ILS may thus result in the wrong conclusion of *P*. *vivax* and *P*. *vivax*-like populations being intermixed and *P*. *vivax* diversity being included in the diversity of *P*. *vivax*-like. A lack of phylogenetic signal, which occurs frequently when species diverged recently, would have similar consequences. In order to test the implication of ILS or lack of phylogenetic signal, we generated a phylogenetic tree and a reticulated network on partial mitochondrial genomes of *P*. *vivax* and *P*. *vivax*-like obtained in Liu and colleagues [10], Prugnolle and colleagues [9], and in the current study. These analyses show that partial mitochondrial genetic information is not enough to make a final conclusion on the origin of *P*. *vivax* parasites (S6 Fig). When considering the polymorphism level of this portion of mitochondrial genomes, over the 2,483 bp, only 127 positions showed variability, 20 of them being a position specific to the outgroup *P*. *cynomolgi*. Among the 107 variable positions found in the *P*. *vivax* and *P*. *vivax*-like samples, 87 were singletons, meaning that only 20 SNVs were shared by more than 2 individuals. This suggests that the discrepancy between our phylogeny and the one of Liu and colleagues [10] is probably because of a lack of phylogenetic signal. Indeed, in our study, the use of significantly more genetic information from throughout the genome, both in genic and intergenic regions, provides a more accurate picture of the genetic relationships between the different parasite species. Reducing our genetic data to single genes (as performed in previous studies) or a limited number of SNVs also generates phylogenies in which *P*. *vivax* is included within the diversity of *P*. *vivax*-like (see S7 Fig).

Another explanation for the discrepancy between our results and the results from Liu and colleagues [10] could be that we are missing part of the diversity of *P*. *vivax*-like given that we obtained the genomes from a limited number of parasites isolated from a small population of apes in Gabon. Nevertheless, this hypothesis does not seem to hold in light of the tree and network produced using all the available mitochondrial sequences of *P*. *vivax*-like and our data, as isolates are distributed all over the currently known genetic diversity of *P*. *vivax*-like (S6 Fig).

Our results show that *P*. *vivax*-like is composed of 2 distinct lineages: one including the 2 reference genomes (Pvl01 and Pvl06) and 7 other isolates that will hereafter be referred to as *P*. *vivax*-like 1, and another one including 2 isolates (Pvl09 and Pvl10) that will hereafter be referred to as *P*. *vivax*-like 2 (Fig 3). This sub-structuration of *P*. *vivax*-like is confirmed in the mitochondrial-reticulated network obtained using a larger number of isolates (see S6 Fig). These 2 lineages may thus reflect an ancient split within *P*. *vivax*-like or be the consequence of a recent introgression or hybridization event between *P*. *vivax*-like and *P*. *vivax* in Africa. A search of recent recombination events between lineages using SplitsTree (http://www.splitstree.org/) [77] does not support this latter hypothesis (S6 Fig).

Previous studies highlighted the high genetic diversity of *P*. *vivax*-like populations in comparison with *P*. *vivax* worldwide [9,10]. In this genome-wide analysis of nucleotide diversity π, we confirm that *P*. *vivax*-like populations are significantly more diverse than *P*. *vivax* populations (*P* < 0.001; Wilcoxon test), with *P*. *vivax*-like samples showing nearly 10 times higher nucleotide diversity (π_*P*.*vivax*_ = 0.0012; π_*P*.*vivax-like*_ = 0.0096). Such genetic diversity in *P*. *vivax*-like strains in comparison with human *P*. *vivax* has already been described in other studies [9,10], suggesting that *P*. *vivax*-like strains probably display extremely high genetic diversity in Central African regions. This suggests that *P*. *vivax*-like parasites of African great apes are probably more ancient than the human *P*. *vivax* strains and that the human *P*. *vivax* species (as for human *Plasmodium* species like *P*. *falciparum*) went through a bottleneck and only recently underwent population expansion.

## A still uncertain origin of *P*. *vivax*

Before the discovery of the ape *P*. *vivax*-like, the main hypothetical scenario concerning the origin of *P*. *vivax* was that of an “Out of Asia.” More specifically, it was considered that *P*. *vivax* emerged in humans following its transfer to humans from Asian monkeys, as has been recently described for *P*. *knowlesi* [42]. However, the recent discovery of *P*. *vivax*-like in African great apes [10] and the analysis of their genetic characteristics led researchers to propose an “Out of Africa” origin of *P*. *vivax* [10,36]. Based on phylogenetic analyses of partial mitochondrial genomes and nuclear sequences of *P*. *vivax* and *P*. *vivax*-like parasites isolated from great apes and humans, Liu and colleagues [10] suggested that all extant human *P*. *vivax* parasites derived from one single ancestor that was transferred from great apes to humans.

Our results do not bring any new evidence in favor of one or the other scenario. We think, nevertheless, that the origin of *P*. *vivax* is more complex than recently proposed (a single host switch from apes to humans in Africa) and that some previous data—especially those regarding the paraphyly of *P*. *vivax*-like and the inclusion of *P*. *vivax* within the diversity of *P*. *vivax*-like (the arguments used in support of an African scenario)—have been overinterpreted. The inclusion of *P*. *vivax* into the *P*. *vivax*-like diversity based on only a couple of nuclear genes and partial mitochondrial genomes is not a definitive proof of transfer of *P*. *vivax*-like to humans in Africa because alternative explanations can be provided. Indeed, as discussed above, such a phylogenetic pattern can be obtained because of ILS or because of a lack of phylogenetic signal for the sequences used, 2 phenomena that are frequent when species diverged recently (which is the case for *P*. *vivax* and *P*. *vivax*-like). Our study indeed shows that incongruent phylogenies may be obtained when one limits the analyses to a single or a couple of genes (see section above and S6 and S7 Figs).

In our mind, it is still impossible to conclude that *P*. *vivax* has an African origin and that it was transferred from African apes to humans, especially when several observations are more in favor of an Asian origin, such as the following: (i) *P*. *vivax* evolved in a clade of parasites infecting Asian monkeys, and (ii) the highest genetic diversity of *P*. *vivax* is observed in Asian populations, and its diversity decreases toward Africa. This pattern of diversity is the opposite for *P*. *falciparum*, which has a well-established African origin [43]. For this parasite, the highest genetic diversity is found in Africa and decreases toward Asia accompanying the human migration [43].

Because the data that are currently available are not easy to interpret and are somehow contradictory, we think that more complex scenarios regarding the origin of *P*. *vivax* should be envisaged in light of phylogenetic and population genetic evidences (as proposed in Prugnolle and colleagues [9]) and more genomic data need to be obtained in support of these scenarios.

## *P*. *vivax*-specific adaptive evolution

Comparison of the *P*. *vivax* genome to its closest sister lineage (*P*. *vivax*-like) and to the other primate *Plasmodium* parasites provides a unique opportunity to identify *P*. *vivax*–specific adaptations to humans. We applied a branch-site test of positive selection to detect events of positive selection that exclusively occurred in the *P*. *vivax* lineage. Within the reference genome *P*. *vivax*-like (Pvl01), 418 genes exhibited significant signals of positive selection (S5 Table). In the human *P*. *vivax* genome PvP01, the test allowed the identification of 255 genes showing significant signals of positive selection (S6 Table). Among these genes presenting a significant *d*_N_/*d*_S_ ratio, 71 were shared between *P*. *vivax* and *P*. *vivax*-like (genes indicated in orange in the S6 Table), including 56 encoding for proteins with unknown function and 15 encoding for proteins that are involved either in energy metabolism regulation (*n* = 9), chromatid segregation (*n* = 2), or cellular-based movement (*n* = 4).

We then took into consideration those 255 genes detected under positive selection in *P*. *vivax* and compared them to those obtained in *P*. *falciparum* (172 genes under selection; see Supplementary Table 4 in [13]). We identified a subset of 10 genes under positive selection in both the human *P*. *vivax* and *P*. *falciparum* parasites (*P* < 0.05) (S7 Table). Among these 10 genes, 5 code for conserved *Plasmodium* proteins with unknown function and 3 for proteins involved in either transcription or transduction. Interestingly, the 2 remaining genes under positive selection in these 2 human *Plasmodium* parasites code for the oocyst capsule protein, which is essential for malaria parasite survival in the *Anopheles*’ midgut, and for the rhoptry protein ROP14, involved in protein maturation and the host cell invasion. These results suggest that these proteins could be essential for infection of humans or their vectors, and future studies should focus on the involvement of these proteins in human parasite transmission and infection.

## Conclusion

Through technical accomplishments, we produced and assembled the first *P*. *vivax*-like reference genomes, the closest sister clade to human *P*. *vivax*—an indispensable step for a better understanding of this enigmatic species. We established that *P*. *vivax*-like parasites form a genetically distinct clade from *P*. *vivax*. Concerning the relative divergence dating, we estimated that the divergence between both species occurred probably before the split between *P*. *malariae* species. This suggests that the transfer of *Plasmodium* parasites to humans happened several times independently over the history of the *Homo* genus. Our genome-wide analyses provided new insights into the adaptive evolution of *P*. *vivax*. Indeed, we identified several key genes that exhibit signatures of positive selection exclusively in the human *P*. *vivax* parasites and show that some gene families important for RBC invasion have undergone species-specific evolution in the human parasite, e.g., *rbp* and *dbp*. Are these genes the keys to the emergence of *P*. *vivax* in the human populations? This pending question will need to be answered through functional studies associated with deeper whole-genome analyses. Among the genes identified to be under positive selection, 2 were also identified to be under positive selection in the other main human malaria agent, *P*. *falciparum*, thus suggesting their key role in the evolution of the parasites in their ability to infect humans or their anthropophilic vectors. To conclude, this study provides the foundation for further investigation into *P*. *vivax* traits that are of public health importance, such as features involved in host–parasite interactions, host specificity, and species-specific adaptations.

## Materials and methods

### *P*. *vivax*-like sample collection and preparation

*P*. *vivax*-like samples were identified by molecular diagnostic testing during a continuous survey of great ape *Plasmodium* infections carried out in the Park of La Lékédi, in Gabon, by the Centre International de Recherches Médicales de Franceville (CIRMF) [9]. In parallel, a survey of *Anopheles* mosquitoes circulating in the same area was conducted in order to identify potential vectors of ape *Plasmodium* [11]. Specifically, mosquitoes were trapped with CDC light traps in the forest of the Park of La Lékédi. *Anopheles* specimens were retrieved and identified using a taxonomic key [44] before proceeding to dissection to isolate the abdomen. Samples were then stored at −20 °C until transportation to the CIRMF, Gabon, where they were stored at −80 °C until processed. Blood samples of great apes were treated using leukocyte depletion by CF11 cellulose column filtration [45]. *P*. *vivax*-like samples were identified either by amplifying and sequencing the *Plasmodium Cytochrome b* (*Cytb*) gene as described in Ollomo and colleagues [46] or directly from samples already used for studies of other *Plasmodium* species [13,14]. This allowed the detection of 11 *P*. *vivax*-like samples, 10 from chimpanzees and 1 from an *An*. *moucheti* mosquito. Most of these samples were co-infected with other *Plasmodium* species and/or probably with multiple *P*. *vivax*-like isolates (see below and S1 Table). The identification of intraspecific *P*. *vivax*-like co-infections was made by analyzing the distribution of the RAF [47].

### Ethical approval

The animals’ well-being was guaranteed by the veterinarians of the “Parc of La Lékédi” and the CIRMF, who were responsible for the proceeding sanitary procedures (including blood collection). All animal work was indeed conducted according to relevant national and international guidelines. These investigations were approved by the Government of the Republic of Gabon and by the Animal Life Administration of Libreville, Gabon (no. CITES 00956). It should be noted that our study did not involve randomization or blinding.

### Genome sequencing

To overcome host DNA contamination, within 6 hours after blood collection in the La Lékédi park, each 5 mL chimpanzee blood sample was diluted with 1 volume of PBS and passed through CF11 cellulose powder columns to remove host leukocytes (i.e., leukocyte depletion) [45]. DNA was then extracted for the 11 samples using Qiagen Midi extraction kits (Qiagen) following the manufacturer’s recommendations. Next, DNA samples were enriched using a WGA using REPLI-g Mini Kit (Qiagen) following the strategy described in Oyola and colleagues [48] to optimize WGA of AT-rich genomes from low DNA quantities. The Illumina isolates were sequenced using Illumina Standard libraries of 200- to 300-bp fragments, and amplification-free libraries of 400- to 600-bp fragments were prepared and sequenced on the Illumina HiSeq 2500 and the MiSeq version 2 according to the manufacturer’s standard protocol (S1 Table). Because of its high DNA content, the Pvl06 isolate was sequenced using PacBio chemistry. After a greater than 8-kb–size selection of DNA fragments using the BluePippin Size-Selection System (Sage Science), the library for this Pvl06 sample was sequenced using P6 polymerase and PacBio chemistry version C3/P5 in 20 SMRT cells. Raw sequence data are deposited in the European Nucleotide Archive. The accession numbers can be found in S1 Table.

### Assembly of *P*. *vivax*-like genomes

#### Host DNA decontamination

To overcome host DNA contamination for the chimpanzee blood samples, contigs obtained were compared to chimpanzee or *An*. *gambiae* genomes (CHIMP2.1.4 genome, accession number: GCA_000001405; and AgamP3 genome, accession number: GCA_000005575) using Basic Local Alignment Tool (BLAST). When more than 50% of the contig hit the chimpanzee reference genome with more than 95% identity, the contig was removed from the analysis.

#### Determination of multiple *Plasmodium* species infections

To identify and quantify *Plasmodium* multispecies infections, Illumina reads from each sample were mapped against the reference genomes of the *Plasmodium* parasite species infecting primates *P*. *vivax* PvP01 [15], *P*. *cynomolgi* M version 2 [17], *P*. *knowlesi* H strain [18], *P*. *reichenowi* PrG01, *P*. *billcollinsi* PbilcG01, and *P*. *gaboni* Pgab01 [13] using BWA version 0.7.12 [49] with default options.

#### *P*. *vivax*-like multiple infections

Following Chan and colleagues [47,50], we examined the RAF distributions to evaluate the possibility of co-infections with multiple strains of *P*. *vivax*-like. A U-shape of the RAF distribution—meaning that almost all positions carry either the reference or a single alternate allele (i.e., RAF of 100% or 0%)—would suggest a single infection. In contrast, if we observe both the reference and alternate alleles at some positions, this would suggest the presence of several strains in the sample. For each *P*. *vivax*-like sample, Illumina reads were mapped against the PvP01 [15] and other chimpanzee-infecting *Plasmodium* species *P*. *gaboni*, *P*. *billcollinsi*, and *P*. *reichenowi* reference genomes [13] using BWA version 0.7.12 [49] with default parameters. We only kept properly paired reads mapping to the PvP01 genome and removed PCR duplicates. SNVs were called independently for each sample using Samtools mpileup followed by bcftools version 0.1.19 [51,52] and were filtered to remove positions matching at least one of the following conditions: a quality phred score ≤30, a coverage <20, or a *P* value for strand bias, mapping quality, or tail distance biases ≤0.001. For each sample and each of the filtered positions, we calculated the percentage of reads carrying the reference allele to draw the RAF distributions.

#### *P*. *vivax*-like genome assembly

Two *P*. *vivax*-like genomes (Pvl01 and Pvl06) were assembled from a co-infection with a *P*. *malariae*-like and a *P*. *reichenowi* (PmlGA01 sample in Rutledge and colleagues [2017] [14]) for Pvl01 and from a co-infection with *P*. *gaboni* for Pvl06 (PGABG01 sample in Otto and colleagues [13]). Briefly, the genome assembly of the Illumina-sequenced sample Pvl01 was performed using MaSuRCA [53], and the assembled contigs belonging to *P*. *vivax*-like were extracted using a BLAST search against the *P*. *vivax* P01 reference genome [15]. The draft assembly was further improved by iterative uses of SSPACE [54], GapFiller [55], and IMAGE [56]. The 3,540 contigs resulting from these analyses were then ordered against the PvP01 genome and the *P*. *gaboni* and *P*. *reichenowi* reference genomes [13] to separate possible co-infections with a parasite species of chimpanzees from the *Laverania* subgenus using ABACAS2 [57]. The genome assembly was further improved and annotated using the Companion web server [16]. BLAST searches of the unassembled contigs against the 3 reference genomes were performed before running Companion to keep the contigs with the best BLAST hits against PvP01 only. The PacBio assembly of Pvl06 was performed using the Hierarchical Genome Assembly Process (HGAP) [58].

### Variant calling for additional samples

#### Read mapping and alignment

Nine additional *P*. *vivax*-like samples were sequenced for population genomics and polymorphism analyses (see S1 Table). The dataset was completed with 19 globally sampled *P*. *vivax* isolates [38] for human versus great ape parasite comparisons, and the Asian parasite *P*. *cynomolgi* strain B [8] was used as the root for phylogenetic inferences (see S1 Table). BWA [49] was found to be as specific as reads from *Laverania* samples did not map onto the reference *P*. *vivax* genome PvP01 (Otto, pers. com., results not shown). The 11 newly generated *P*. *vivax*-like samples, together with the already published 19 *P*. *vivax* samples and the reference strain *P*. *cynomolgi* [8] reads, were therefore mapped against the PvP01 reference genome using BWA version 0.7.12 [49] with default parameters. We then used Samtools version 0.1.19 to keep only the properly paired reads and to remove PCR duplicates [49]. Data quality was evaluated by the means of the number of reads properly paired, the mean depth per site, and the proportion of sites covered by at least 1, 5, 10, or 20 reads (see S3 Table). Only positions with a quality phred score ≥30 and a *P* value for strand bias, mapping quality, or tail distance bias >0.001 were considered.

#### SNV discovery

For population genomics and polymorphism analyses, SNVs were called independently for all 11 *P*. *vivax*-like and 19 *P*. *vivax* samples from the sequence alignment files generated using BWA [49] to map the reads against the PvP01 genome (see above) [15]. For the SNV calling, we used Samtools mpileup version 0.1.19 [49] (parameters–q 20 -Q 20 -C 50) followed by bcftools to remove indels (call -c -V indels) [49]. SNVs were filtered using VCFTools [59] to keep variants that have been successfully genotyped in 100% of individuals, with a minimum depth of 5 reads per position. Allelic frequencies were also estimated for each variant at each SNV in order to select the dominant allele in multiple infections (—minDP 5 –max-missing 1—freq). Then, the files in VCF format were transformed into tab-delimited text format, using the “vcf-to-tab” Perl module, and finally transformed into fasta format using a homemade Perl script that could be obtained directly by contacting corresponding authors.

### Gene family search

For the *P*. *vivax*-like Pvl01 and Pvl06, *P*. *vivax* PvP01 and SalI, *P*. *cynomolgi* B strain, and *P*. *knowlesi* H strain genomes, gene variants were detected and counted using Geneious software [60]. This allowed the presence/absence of the variants of the different gene families across *Plasmodium* species to be evaluated. For each gene family, the number of variants identified in the 2 reference genomes Pvl01 and Pvl06 was confirmed by manually checking the presence/absence of these in the other *P*. *vivax*-like genotypes obtained using ACT and bamview [61,62].

### Orthologous group determination and alignment

Orthologous groups across (1) *P*. *vivax* PvP01 [15], *P*. *vivax*-like Pvl01, *P*. *cynomolgi* B strain [8], and *P*. *knowlesi* H strain [18] reference genomes and (2) the 13 *Plasmodium* reference genomes used for the phylogeny (the here-generated *P*. *vivax*-like Pvl01, *P*. *vivax* PvP01 [15], *P*. *cynomolgi* M version 2 [17], *P*. *coatneyi* strain Hackeri [63], *P*. *knowlesi* H strain [18], *P*. *falciparum* 3D7 [64], *P*. *praefalciparum* G01 [13], *P*. *reichenowi* PrCDC [65], *P*. *gallinaceum* 8A [30], *P*. *ovale wallikeri* PowCR01, *P*. *ovale curtisi* PocCR01, *P*. *malariae* PmUG01, and *P*. *malariae*-like *PmlGA01* [14]) were identified using OrthoMCL version 2.09 [66,67] after an all-against-all BLASTp (E-value threshold: 1 × 10^−6^). From these, we extracted different sets of one-to-one orthologous genes for the subsequent analyses: a set of 4,056 genes that included the one-to-one orthologues among the 4 restricted species—*P*. *vivax*, *P*. *vivax*-like, *P*. *cynomolgi*, and *P*. *knowlesi* (the Pv4sp set)—and a set of 2,943 among the 13 *Plasmodium* species considered here for the interspecies phylogenetic analysis (the Pl13sp set). The first set of orthologous groups identified was used for the detection of selection (see below); the second one was used to build the phylogeny and for the dating analyses.

Amino acid sequences of the one-to-one orthologues were aligned using MUSCLE [68] or MAFFT [69], respectively, for the first and second set of orthologous groups. Prior to aligning codon sequences, we removed the low-complexity regions identified on the nucleotide level using dustmasker [70] and then in amino acid sequences using segmasker [71] from ncbi-blast, using default parameters. After MUSCLE/MAFFT alignments, we finally excluded poorly aligned codon regions using Gblocks (parameters: -t = p -b5 = h -p = n -b4 = 2) [72]. After going through all these alignment cleaning steps, we ended with a low number of missing data and no gap in our dataset of one-to-one orthologues (31 missing data over the 2,784 alignments of more than 50 amino acids).

### Divergence dating

Most of the current methods available to estimate the timing of species splits make strong assumptions on the evolutionary models and often require an accurate mutation rate or calibration points, which are not always available. Here, we estimated the relative divergence times of *Plasmodium* species to free from temporal calibration of phylogenies and used 2 methods that do not depend on the estimation of a mutation rate: a method based on pairwise amino acid sequence divergences and Total Least Squares (TLS) regressions but assuming a constant rate of evolution across the *Plasmodium* lineages—the so-called Silva method [29]—and the RelTime method developed in Tamura et al. [31,32], which does not assume any specific model for the rate of evolution.

We first built the phylogenetic tree of the 13 *Plasmodium* species considered here for the dating analyses using the software RAxML version 8.2.8 [73]. The tree was constructed using the concatenation of the 2,784 orthologous groups that have a final alignment of more than 50 amino acids (among the Pl13sp set of 2,943 groups). RAxML was called using the following options in order to automatically determine the substitution model that best fits the data model and so that 100 bootstraps were performed to assess the tree robustness: “-m PROTGAMMAAUTO -f a -# 100.” The best amino acid model of substitution identified by RAxML for the dataset (the JTT model with empirical base frequencies JTT + F) was used in the subsequent analyses.

The idea behind the Silva method [29] for relative age estimation is that the divergence between amino acid sequences in independent lineages is correlated and that the divergence regression slopes of the proteins of a species pair of reference with the divergence of the proteins of other species pairs reflect the relative age of those splits (see Silva and colleagues [29] for a detailed description of the method). Following this method, we obtained, for each of the 2,784 orthologous groups of the Pl13sp set that have a final alignment of more than 50 amino acids and for each species pair, the amino acid sequence divergence *d*_AA_ through a pairwise comparison using PAML version 4.7 [74], with the option “cleandata” set to 1 to remove sites with missing data from the dataset. The sequence divergences *d*_AA_ were estimated using 4 different models of substitution (JTT, WAG, LG, and Dayhoff) to evaluate their influence on the estimates of the relative ages. An R script from the authors of this method [29] allowed the estimation of *α*, the slopes of the TLS regressions of the divergence of the proteins between every possible species pairs and the reference species pair, with the 95% CI by bootstrapping (*n* = 10,000 bootstraps). The slope α is an estimator of the relative age of the 2 considered species, relative to the reference species pair. To evaluate the influence of the choice of the reference species pair on the results, we used different species pairs to consider multiple divergence references: We considered the relative distance of the split of the speciation of (i) *P*. *vivax* and *P*. *knowlesi* (reference pair considered in the original paper), (ii) the 2 *P*. *ovale* species, and (iii) the *P*. *malariae*-like and *P*. *malariae* species.

Because the model of Silva assumes a strict molecular clock [29]—which would not apply to all *Plasmodium* species, specifically *P*. *falciparum* because of its extreme GC content in comparison with other *Plasmodium* species—we used the clock calibration-free method RelTime [31,32] implemented in MEGA7 [75] to estimate divergence times when evolutionary rates vary. RelTime estimates relative rates for each branch of the tree without requiring a distribution of rate heterogeneity. The relative rates of 2 sister lineages are estimated assuming that the time to their most recent common ancestor is equal in the presence of contemporaneous sampling, and the rate of their ancestral branch is estimated as the average of their branch-specific relative rates. This framework is applied to subtrees of 3 or 4 taxa following a bottom-up strategy, from tips to the root to estimate relative rates and times, relative rates being scaled by setting the lineage relative rate of the ingroop root node to 1 (see Tamura and colleagues [32]). RelTime analysis was performed on the concatenation of the alignments of the same 2,784 orthologous groups we used to build the phylogenetic tree, removing sites with missing data (Complete-Deletion option) and using the same substitution model (JTT + F). As required by the RelTime analysis, we specified *P*. *gallinaceum* 8A [30] as the outgroup.

### Phylogenetic tree of *P*. *vivax* and *P*. *vivax*-like strains

The phylogenetic relationships of the RBPs in the *P*. *cynomolgi*–*P*. *knowlesi*–*P*. *vivax*–*P*. *vivax*-like group of species was reconstructed using a maximum likelihood analysis using RAxML [73] with 100 bootstrap replicates. The tree was visualized using Geneious software [60]. To investigate relationships between the *P*. *vivax* and *P*. *vivax*-like populations, we constructed a maximum likelihood tree using the filtered variant call set of SNVs limited to the higher allelic frequency genotypes identified within each sample using RAxML and PhyML (using general time-reversible [GTR] models) [73,76]. Trees were visualized using Geneious software [60]. All approaches showed the same final phylogenetic tree described in the “Relationships to worldwide human *P*. *vivax* isolates” section.

### Analysis of phylogenetic discordance by means of reticulated networks

We looked for signatures of introgression and/or ILS by performing a phylogenetic network using SplitsTree 4.14.6 [77] based on the alignment of a portion of the mitochondrial genome containing the *cox1* and *cytb* genes, as in Liu and colleagues [10]. We included all but 2 (KF618538 and KF618534) samples from Liu and colleagues [10], all from Prugnolle and colleagues [9], and 3 of our samples with a high-quality–sequenced mitochondrial genome (Pvl08, Pvl09, and Pvl10), and we added *P*. *cynomolgi* as the outgroup (GenBank accession number: AB434919.1). Gaps and monomorphic positions were excluded for the analysis, and the dataset resulted in an alignment of 85 sequences of 127 variable positions. We conducted a reticulate network using the RECOMB 2007 [78] and the NeighborNet distance transformation methods [79]. A phylogenetic tree based on the complete alignment, without exclusion of sites (i.e., an alignment of 2,530 bp), was constructed using FastTree 2.1.5 (maximum likelihood) implemented in Geneious [60] for comparison with results obtained from Liu and colleagues [10]. To explore the influence of the amount of phylogenetic signal on phylogenetic reconstruction, we also performed phylogenetic trees as described in the previous section using a subset of SNVs: Trees were reconstructed using 100, 200, 300, 400, 500, 600, 800, 1,000, or 5,000 SNVs.

### Genome-wide nucleotide diversity

The nucleotide diversity (π) is the average number of nucleotide differences per site between 2 sequences. This parameter is interesting to estimate because it gives valuable information about variation in prevalence and demographic histories of the parasites. For the *P*. *vivax* and *P*. *vivax*-like populations, we calculated the genome-wide nucleotide diversity (π) from VCF files using VCFTools [59]. The means of nucleotide diversities was compared between *P*. *vivax* and *P*. *vivax*-like species based on the Wilcoxon-Mann-Whitney nonparametric test.

### Detection of genes under selection

In order to identify genomic regions involved in parasite adaptation to the human host, i.e., regions under positive selection, we performed branch-site tests. To search for genes that have been subjected to positive selection in the *P*. *vivax* lineage alone, after the divergence from *P*. *vivax*-like, we used the updated branch-site test of positive selection [80] implemented in the package PAML version 4.4c [74]. This test detects sites that have undergone positive selection in a specific branch of the phylogenetic tree (foreground branch) using Likelihood Ratio Tests (LRTs) to compare models allowing positive selection or not. The selective pressure is defined as the ratio (*ω*) of nonsynonymous (i.e., *d*_N_, keeping the amino acid) to synonymous (i.e., *d*_S_, changing the amino acid) substitutions (*d*_N_/*d*_S_). Under neutrality, rates of synonymous *d*_S_ and nonsynonymous *d*_N_ substitutions are equivalent, so the *ω* ratio is expected to be equal to 1. Under purifying selection, the rate of nonsynonymous substitutions *d*_N_ is expected to be lower than the rate of synonymous substitution *d*_S_ because selection will prevent amino acid modifications: The *ω* ratio observed is then under 1. This process is acting in reducing the fixation of deleterious mutation. Finally, under positive selection, amino acid replacement will be chosen, with the *d*_N_ rate expected to be superior to the *d*_S_ rate, leading to an observed ω ratio superior to 1. This indicates the fixation of advantageous mutations. All coding sequences in the core genome with a one-to-one orthologous relationship among *P*. *vivax* Sal1, *P*. *vivax*-like Pvl01, *P*. *knowlesi* H strain, and *P*. *cynomolgi* B strain were used for this test (i.e., the 4,056 gene Pv4sp set of orthologous genes). We obtained *d*_N_/*d*_S_ ratio estimates per branch and gene for *P*. *vivax* and *P*. *vivax*-like lineages alone using Codeml (PAML version 4.4c [74]) with a free-ratio model of evolution. Genes with a significant signal of positive selection in *P*. *vivax* only were compared to the ones obtained in *P*. *falciparum* from Otto and colleagues [13] (S4 Table in Otto and colleagues [13]), in order to identify, e.g., essential proteins for human or vector infection.

The data are deposited in the Dryad repository (doi:10.5061/dryad.32tm1k4) [81].

## Supporting information

S1 Table Overview of the *P*. *vivax*-like and *P*. *vivax* samples. *Plasmodium* species, sample ID, accession number, geographic location, year of collection, *Plasmodium* co-infections, host species infected by this *Plasmodium* parasite, bibliographic reference, and usage are indicated. For multispecies co-infections, a sample was considered infected with a *Plasmodium* species when the percentage of reads mapping to the reference genome of that species was >3% (see S2 Table and Materials and methods section).
(XLSX)

S2 Table Sequencing and *Plasmodium* multispecies co-infections information. Host contamination was estimated by mapping the reads against the human genome from the Genome Reference Consortium GRCh37 (Genbank accession: GCA_000001405) for all samples but Pvl09, for which reads were mapped against the reference genome of *An*. *gambiae* str. PEST (Genbank accession: GCA_000005575). Those reads mapping the human or the mosquito genomes were discarded. *P*. *vivax*-like samples were analyzed for multispecies infections by mapping reads against the reference genome of chimpanzee-infecting *Plasmodium* parasites.
(XLSX)

S3 Table Overview of the SNV calling of the *P*. *vivax*-like samples. *p1, p5, p10, and p20 refer to the percentage of positions covered by at least 1, 5, 10, or 20 reads, after filtering to select high-quality positions (quality phred score ≥30 and a *P* value for strand bias, mapping quality, or tail distance bias >0.001; see Materials and methods section for additional information). SNV, single nucleotide variant.
(XLSX)

S4 Table Overview of the dating analyses under the JTT model of evolution. (A) Influence of the choice of the species pair of reference for the divergence dating method of Silva and colleagues [29]. The slopes of the regressions *α* of the protein divergence between each species pair and the protein divergence between the reference species pairs (representing the relative age of the species pair of interest relative to that of the reference pair considered) and the 95% CI are given in columns C–O. The ratio of *α* measured considering PocGH01-PowCR01 or PmUG01-PmlGA01 as the reference pair to α measured considering PKNH-PvP01 as the reference pair is given for each species pair, as well as the mean over the species pairs, the SD, and the coefficient of variation (SD/mean), in columns Q and R, respectively. (B) Relative divergence dates estimated using RelTime [31,32]. PVL, *P*. *vivax*-like Pvl01; PvP01, *P*. *vivax* PvP01; PcyM, *P*. *cynomolgi* M; PCOAT2, *P*. *coatneyi* strain Hackeri; PKNH, *P*. *knowlesi* strain H; PowCR01, *P*. *ovale wallikeri* CR01; PocCR01, *P*. *ovale curtisi* CR01; PmUG01, *P*. *malariae* UG01; PmlGA01, *P*. *malariae*-like GA01; PF3D7, *P*. *falciparum* 3D7; PPRFG01, *P*. *praefalciparum* G01; PRCDC: *P*. *reichenowi* CDC; PGAL8A, *P*. *gallinaceum* 8A.
(XLSX)

S5 Table *P*. *vivax*-like PVL01 genome-wide signatures of selection. The branch-site test of positive selection for orthologous genes product, gene length, *d*_N_/*d*_S_ ratio for PVL01 as well as the *P* value for this test are indicated. Only significant tests are reported. The test was considered significant when the *P* value was below 0.05. In orange are indicated genes with significant *d*_N_/*d*_S_ ratios between *P*. *vivax* PvP01 and *P*. *vivax*-like PVL01. *d*_N_/*d*_S_ = 999 when no synonymous difference is observed in the alignment of the gene considered and thus the ratio is estimated to be infinite.
(XLSX)

S6 Table *P*. *vivax* PvP01 genome-wide signatures of selection. The branch-site test of positive selection for orthologous genes product, gene length, and *d*_N_/*d*_S_ ratio for PvP01, as well as the *P* value for this test, are indicated. Only significant tests are reported. The test was considered significant when the *P* value was below 0.05. In orange are indicated genes with significant *d*_N_/*d*_S_ ratios between *P*. *vivax* PvP01 and *P*. *vivax*-like PVL01. *d*_N_/*d*_S_ = 999 when no synonymous difference is observed in the alignment of the gene considered and thus the ratio is estimated to be infinite.
(XLSX)

S7 Table *P*. *vivax* and *P*. *falciparum* genome-wide signatures of selection. The list of the 10 orthologous genes showing a signature of selection in both *P*. *vivax* PvP01 and *P*. *falciparum* 3D7 is indicated, based on the branch-site tests of positive selection. The branch-site tests of selection for PF3D7 genes were performed by Otto and colleagues [13] (see Suppl. Table 4 from Otto and colleagues [13]); PF3D7 orthologous genes to PvP01 genes were identified using the PlasmoDB database (http://plasmodb.org/plasmo/). *d*_N_/*d*_S_ = 999 when no synonymous difference is observed in the alignment of the gene considered and thus the ratio is estimated to be infinite.
(XLSX)

S1 Fig RAF distributions in the 11 *P*. *vivax*-like samples sequenced in this study. This graph represents, for each sample, the density distribution of the frequency of the reference alleles in the sequenced reads across all sites (x-axis). A U-shape of the RAF distribution, meaning that almost all positions carry either the reference or a single alternate allele (i.e., RAF of 100% or 0%), would suggest a single infection. By contrast, if we observe both the reference and alternate alleles at some positions, this would suggest the presence of several strains in the sample. The data are available in the Dryad repository: doi:10.5061/dryad.32tm1k4. RAF, reference allele frequency.
(PDF)

S2 Fig Genome synteny between *P*. *vivax* reference genome PvP01 and *P*. *vivax*-like genome Pvl01. Top: reference chromosome from *P*. *vivax* PvP01; bottom: Pvl01 genome. Orange = forward strand gene; blue = reverse strand gene; green = missing core gene; black = singleton gene; yellow = gap. Genome annotation stored as embl files, one for each chromosome is available at the Dryad Repository: https://datadryad.org/resource/doi:10.5061/dryad.32tm1k4.2.
(PDF)

S3 Fig Genome synteny between *P*. *vivax* reference genome PvP01 and *P*. *vivax*-like genome Pvl06. Top: reference chromosome from *Plasmodium vivax* PvP01; bottom: Pvl06 genome. Orange = forward strand gene; blue = reverse strand gene; green = missing core gene; black = singleton gene; yellow = gap. Genome annotation stored as embl files, one for each chromosome is available at the Dryad Repository: https://datadryad.org/resource/doi:10.5061/dryad.32tm1k4.2.
(PDF)

S4 Fig Phylogenetic tree, inferred using RAxML program, of *clag* genes in *P*. *vivax* PvP01 (in green), *P*. *vivax*-like Pvl01 (in blue), *P*. *cynomolgi* (B, Berok strain) and *P*. *knowlesi* (H strain). Bootstrap values are indicated. Pictograms represent the host species (human, gorilla, chimpanzee, Asian monkey). The black star indicates a pseudogene detected in pvCLAG8 gene of *P*. *vivax*-like Pvl01. The alignment of the *clag* genes and the resulting tree as inferred here are available as supplemental files in S1 Data.
(PDF)

S5 Fig Influence of the model of substitution on the estimates of the relative divergence times obtained using the method from Silva and colleagues [29]. The values of *α*, the estimations of the divergence time of every species pair relative to that of the species pair of reference (i.e., *P*. *knowlesi*–*P*. *vivax* [Pkn–Pv], *P*. *malariae*–*P*. *malariae*-like [Pm–Pml], or *P*. *ovale curtisi*–*P*. *ovale wallikeri* [Poc–Pow]), estimated under the JTT model of evolution (x-axes) are plotted against the values estimated under the WAG (red), LG (green), and Dayhoff (blue) models of substitution (y-axes). Data can be found in S4 Data.
(PDF)

S6 Fig Relationships between *P*. *vivax* and *P*. *vivax*-like samples based on a portion of the mitochondrial genome. (A) Phylogenetic tree based on an alignment of 2,530 bp of the mitochondrial genome and performed using FastTree 2.1.5 implemented in Geneious [55]. FastTree support values >0.7 are given. (B) Reticulate network built with SplitsTree4 on 126 variable positions located on the mitochondrial genes COX1 and CYTB. The outgroup *P*. *cynomolgi* is indicated with a red square, the human *P*. *vivax* sequences are represented by grey squares, and the ape-infecting species *P*. *vivax*-like by light green, brown, and green squares when isolated from a gorilla, chimpanzee, or *Anopheles* host. The sequences generated during this study are indicated by blue arrows. The alignment of 2,530 bp from the mitochondrial genome used to produce the mitochondrial tree and the dataset for the reticulate network (see Materials and methods for additional information on the generation of this alignment) and the resulting tree are available as supplemental files in S6 Data.
(PDF)

S7 Fig Relationships between *P*. *vivax* and *P*. *vivax*-like samples using phylogenetic trees based on 100, 200, 300, 400, 500, 600, 800, 1,000, or 5,000 SNVs. Data can be found in S7 Data. SNV, single nucleotide variant.
(PDF)

S1 Data Alignment and tree files of the *clag* sequences analyzed in S4 Fig. ***clag*,** Cytoadherence-linked asexual gene.
(GZ) Click here for additional data file.

S2 Data Alignment and phylogenetic tree files of the *rbp* gene sequences analyzed in Fig 1. *rbp*, reticulocyte-binding protein.
(GZ) Click here for additional data file.

S3 Data Alignment used to generate the ML phylogenetic tree of the 13 *Plasmodium* species considered for the dating analyses and the resulting tree file. ML, maximum likelihood.
(GZ) Click here for additional data file.

S4 Data Data used to generate S5 Fig. The table gives the estimations of α, the slopes of the TLS regressions of the divergence of the proteins between every possible species pairs and the reference species pair. We performed the analyses considering 3 different pairs of reference species (column entitled "ref") and 4 different amino acid models of substitution (JTT, WAG, LG, and Dayhoff). Estimates of α are given for every possible species pairs in the phylogenetic tree (column "comp"). PVL, *P*. *vivax*-like Pvl01; PVP01, *P*. *vivax* PvP01; PcyM, *P*. *cynomolgi* M; PCOAT2, *P*. *coatneyi* strain Hackeri; PKNH, *P*. *knowlesi* strain H; PowCR01, *P*. *ovale wallikeri* CR01; PocCR01, *P*. *ovale curtisi* CR01; PmUG01, *P*. *malariae* UG01; PmlGA01, *P*. *malariae*-like GA01; PF3D7, *P*. *falciparum* 3D7; PPRFG01, *P*. *praefalciparum* G01; PRCDC, *P*. *reichenowi* CDC; PGAL8A, *P*. *gallinaceum* 8A.
(TXT)

S5 Data Alignment of the 100,616 SNVs detected among the 11 chimpanzee *P*. *vivax*-like and 19 human *P*. *vivax* samples analyzed in this study. Sample code are the ones used in S1 Table. SNV, single nucleotide variant.
(FASTA)

S6 Data Dataset used to generate S6 Fig. Both the complete alignment of the mitochondrial portion used to reconstruct the phylogenetic tree and the alignment of the 127 variable positions used to perform the reticulate network are given, as well as the resulting tree and network files.
(GZ) Click here for additional data file.

S7 Data Dataset used to produce S7 Fig. For each analysis with a different number of SNVs considered, the alignment and the resulting tree file are available. SNV, single nucleotide variant.
(ZIP)

## Abbreviations

BLAST: Basic Local Alignment Tool
CI: confidence interval
CIRMF: Centre International de Recherches Médicales de Franceville
CLAG: Cytoadherence-linked asexual gene
*Cytb*: *Plasmodium Cytochrome b*
DARC: Duffy Antigen Receptor for Chemokine
DBP: Duffy-binding protein
ETRAMP: early transcribed membrane protein
GC: guanine–cytosine
G-PhoCS: Generalized Phylogenetic Coalescent Sampler
GTR: general time-reversible
HGAP: Hierarchical Genome Assembly Process
ILS: Incomplete Lineage Sorting
JTT: Jones, Taylor, and Thornton
LRT: likelihood ratio test
MCMC: Markov Chain Monte Carlo
PacBio: Pacific Biosciences
PHIST: *Plasmodium* helical interspersed subtelomeric
PIR: *Plasmodium* interspersed repeat
RAF: reference allele frequency
RBC: red blood cell
RBP: reticulocyte-binding protein
SERA: serine-repeat antigen
SNV: single nucleotide variant
STP1: subtelomeric protein 1
TLS: Total Least Squares
TRAG: tryp-rich antigen
WGA: whole-genome amplification
