# Supplementary figures and images for: Plasmodium vivax-like genome sequences shed new insights into Plasmodium vivax biology and evolution
Source: PLoS Biol. 2018 Aug 24;16(8):e2006035. doi: 10.1371/journal.pbio.2006035 (PMC6130868; doi:10.1371/journal.pbio.2006035)

Reference Allele Frequency densities

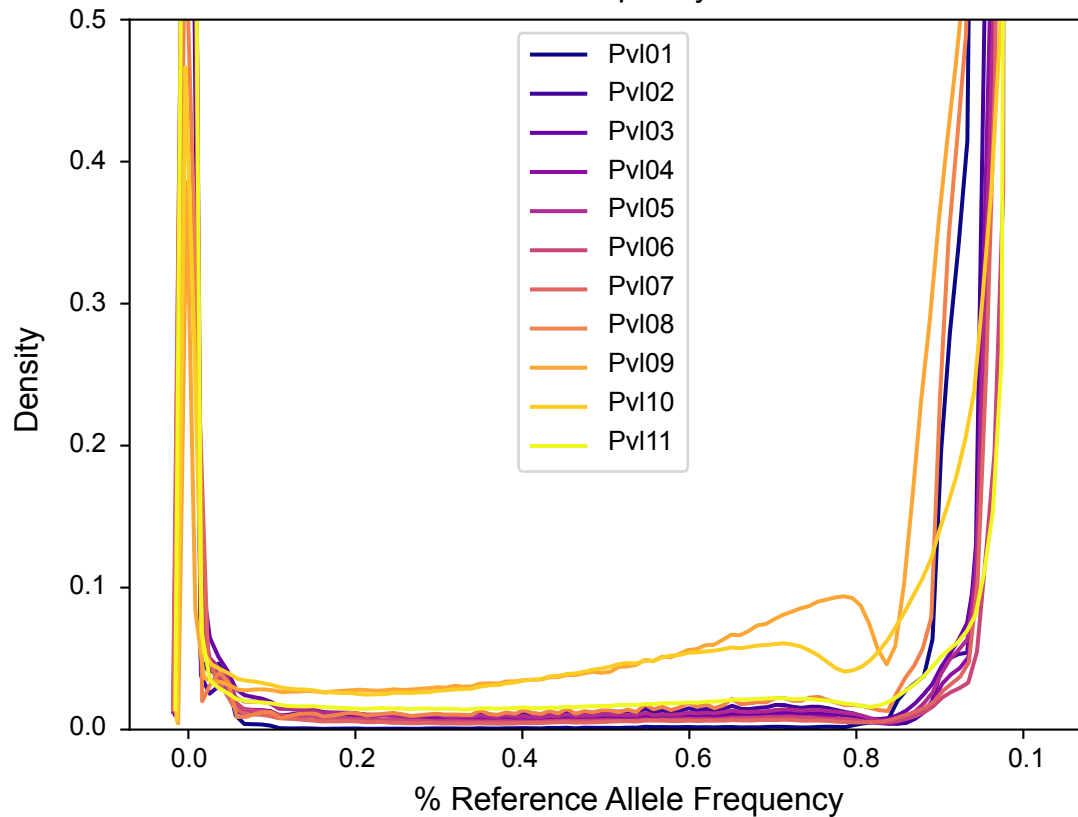

Supplement: S1 Fig — This graph represents, for each sample, the density distribution of the frequency of the reference alleles in the sequenced reads across all sites (x-axis). A U-shape of the RAF distribution, meaning that almost all positions carry either the reference or a single alternate allele (i.e., RAF of 100% or 0%), would suggest a single infection. By contrast, if we observe both the reference and alternate alleles at some positions, this would suggest the presence of several strains in the sample. The data are available in the Dryad repository: doi:10.5061/dryad.32tm1k4. RAF, reference allele frequency. (PDF) [file pbio.2006035.s008.pdf]

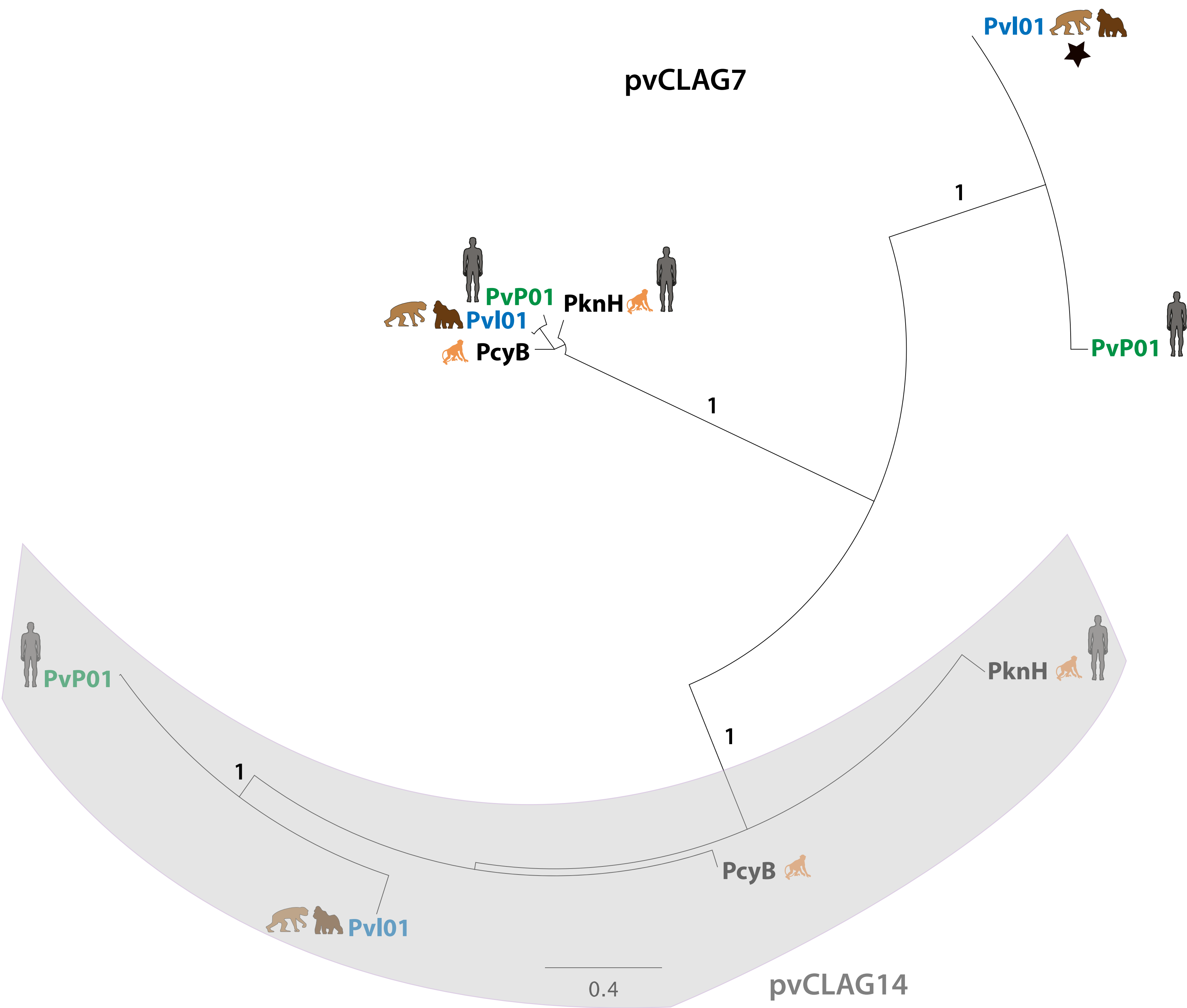

Supplement: S4 Fig — Bootstrap values are indicated. Pictograms represent the host species (human, gorilla, chimpanzee, Asian monkey). The black star indicates a pseudogene detected in pvCLAG8 gene of P. vivax-like Pvl01. The alignment of the clag genes and the resulting tree as inferred here are available as supplemental files in S1 Data. (PDF) [file pbio.2006035.s011.pdf]

# Estimations of $\alpha$

Alternative models

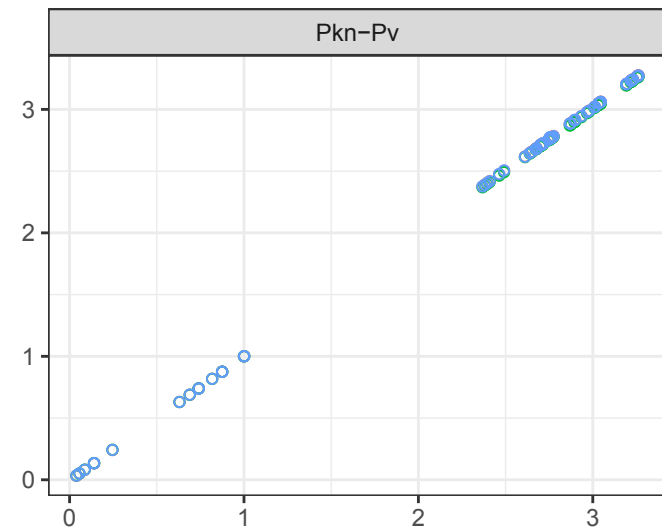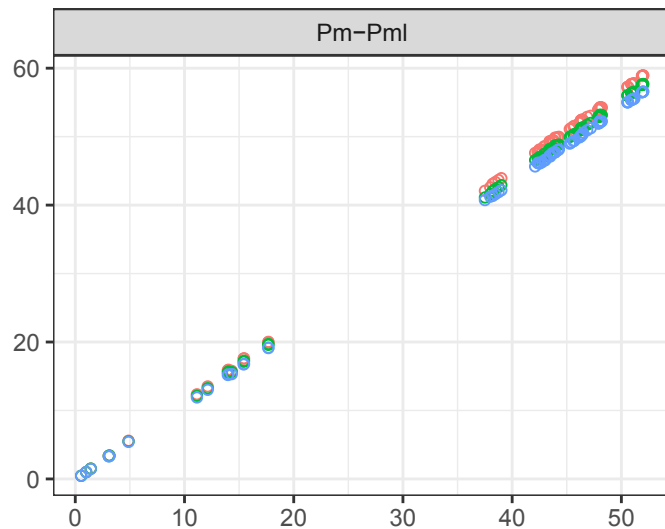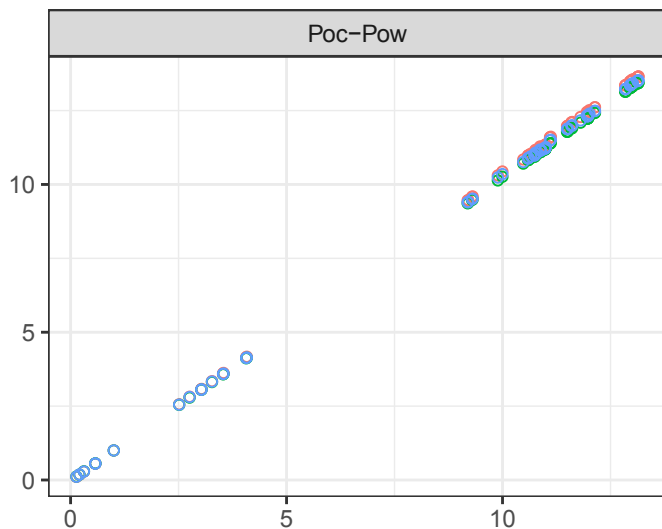

Alternative model

○ WAG

○ LG

○ Dayhoff

Jones model

Supplement: S5 Fig — The values of α, the estimations of the divergence time of every species pair relative to that of the species pair of reference (i.e., P. knowlesi–P. vivax [Pkn–Pv], P. malariae–P. malariae-like [Pm–Pml], or P. ovale curtisi–P. ovale wallikeri [Poc–Pow]), estimated under the JTT model of evolution (x-axes) are plotted against the values estimated under the WAG (red), LG (green), and Dayhoff (blue) models of substitution (y-axes). Data can be found in S4 Data. (PDF) [file pbio.2006035.s012.pdf]

A)

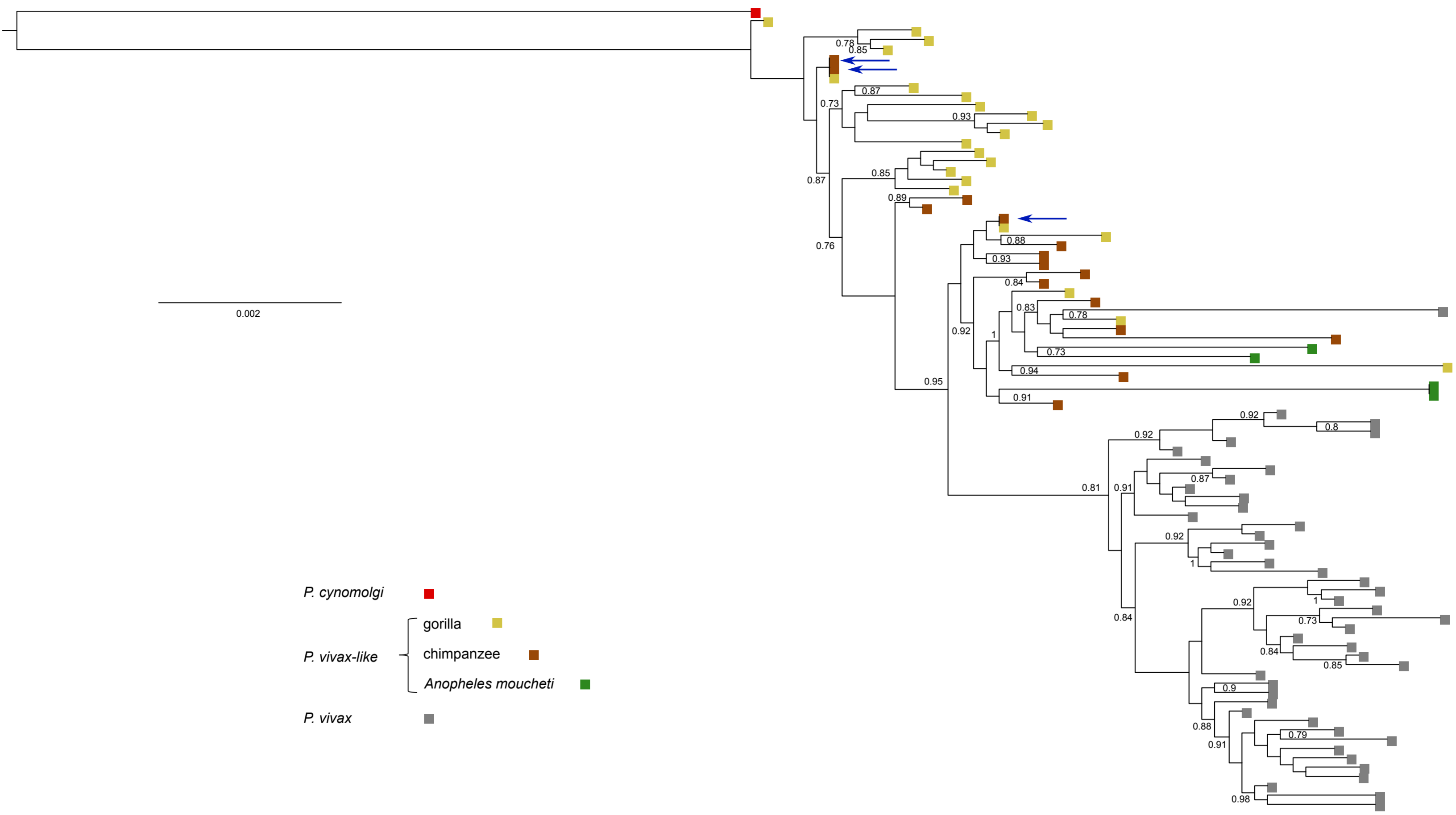

B)

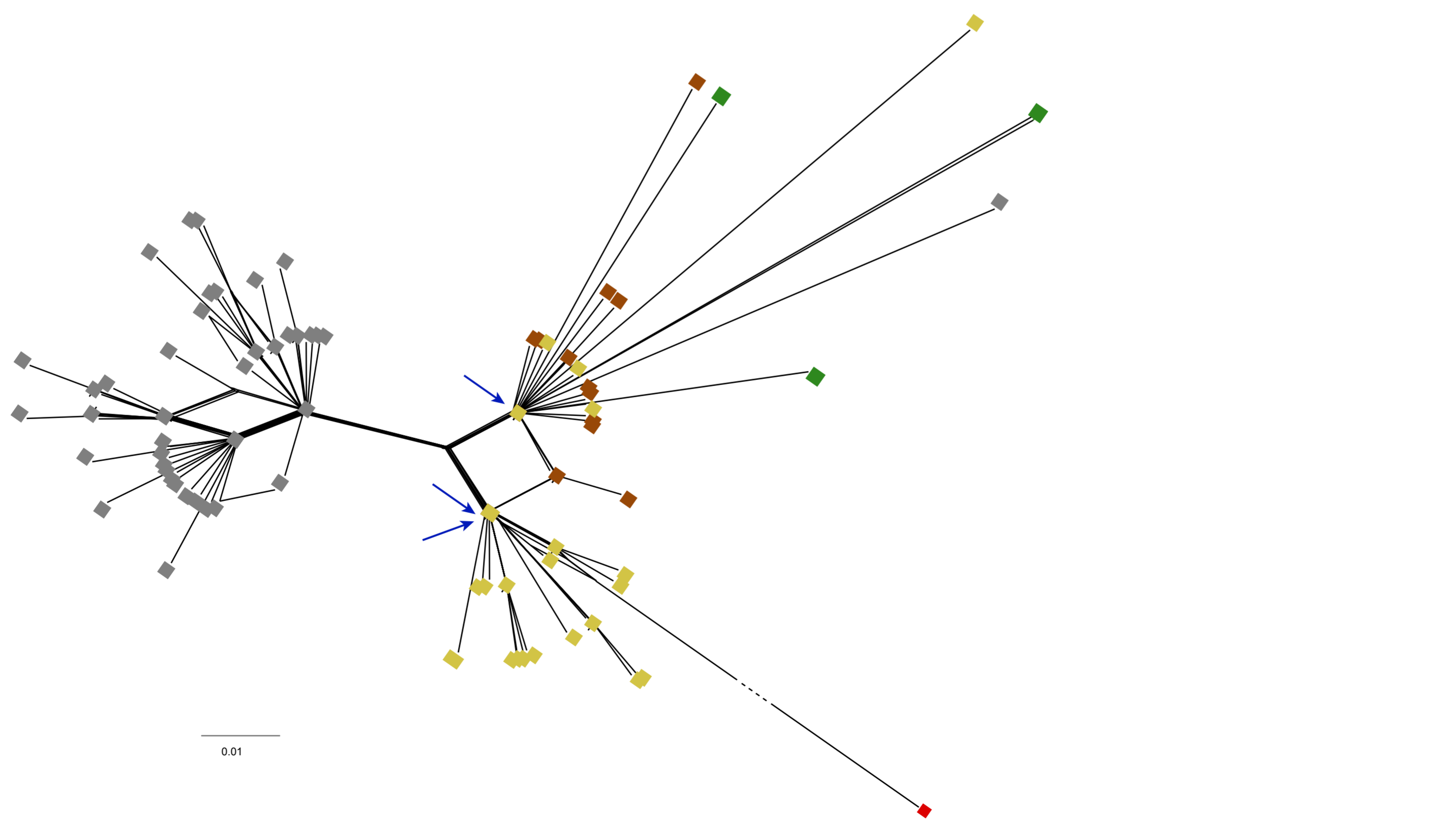

Supplement: S6 Fig — (A) Phylogenetic tree based on an alignment of 2,530 bp of the mitochondrial genome and performed using FastTree 2.1.5 implemented in Geneious [55]. FastTree support values >0.7 are given. (B) Reticulate network built with SplitsTree4 on 126 variable positions located on the mitochondrial genes COX1 and CYTB. The outgroup P. cynomolgi is indicated with a red square, the human P. vivax sequences are represented by grey squares, and the ape-infecting species P. vivax-like by light green, brown, and green squares when isolated from a gorilla, chimpanzee, or Anopheles host. The sequences generated during this study are indicated by blue arrows. The alignment of 2,530 bp from the mitochondrial genome used to produce the mitochondrial tree and the dataset for the reticulate network (see Materials and methods for additional information on the generation of this alignment) and the resulting tree are available as supplemental files in S6 Data. (PDF) [file pbio.2006035.s013.pdf]

100 SNPs

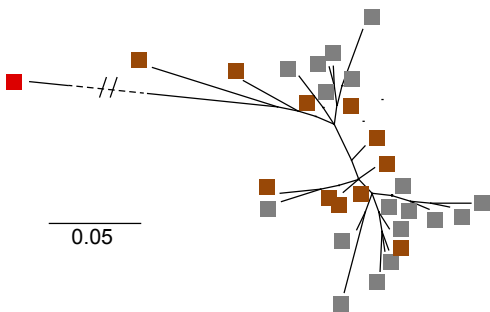

200 SNPs

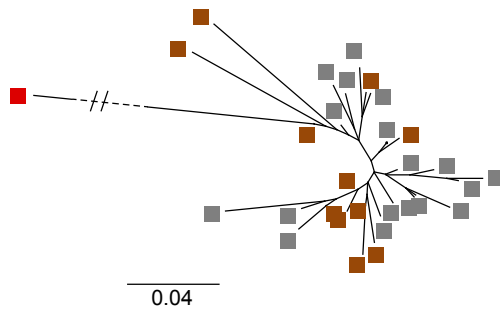

300 SNPs

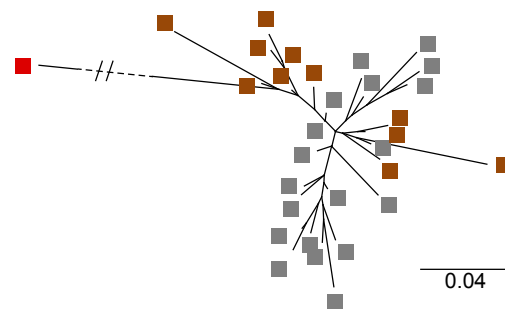

400 SNPs

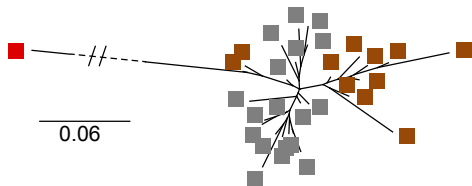

500 SNPs

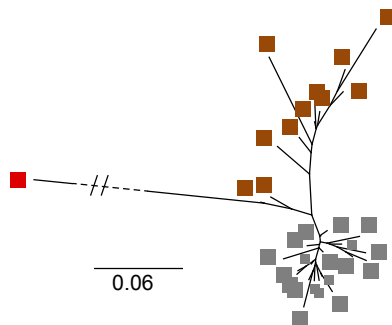

600 SNPs

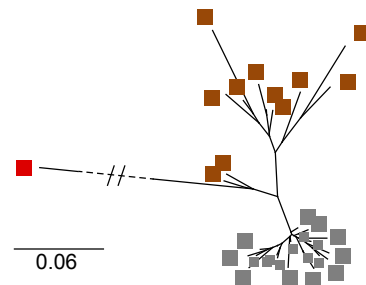

800 SNPs

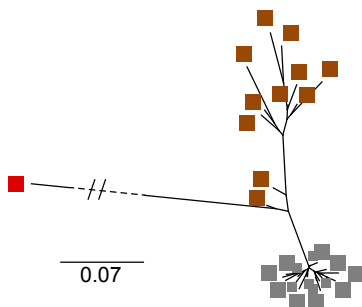

1,000 SNPs

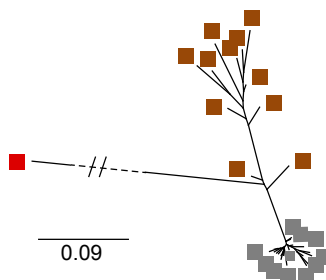

5,000 SNPs

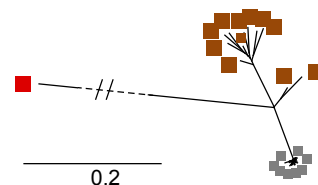*P. cynomolgi* ■*P. vivax-like* ■*P. vivax* ■

Supplement: S7 Fig — Data can be found in S7 Data. SNV, single nucleotide variant. (PDF) [file pbio.2006035.s014.pdf]
