# Supplementary material for: Plasmodium vivax-like genome sequences shed new insights into Plasmodium vivax biology and evolution
Source: PLoS Biol. 2018 Aug 24;16(8):e2006035. doi: 10.1371/journal.pbio.2006035 (PMC6130868; doi:10.1371/journal.pbio.2006035)
Supplement: S2 Fig — Top: reference chromosome from P. vivax PvP01; bottom: Pvl01 genome. Orange = forward strand gene; blue = reverse strand gene; green = missing core gene; black = singleton gene; yellow = gap. Genome annotation stored as embl files, one for each chromosome is available at the Dryad Repository: https://datadryad.org/resource/doi:10.5061/dryad.32tm1k4.2. (PDF) [file pbio.2006035.s009.pdf]

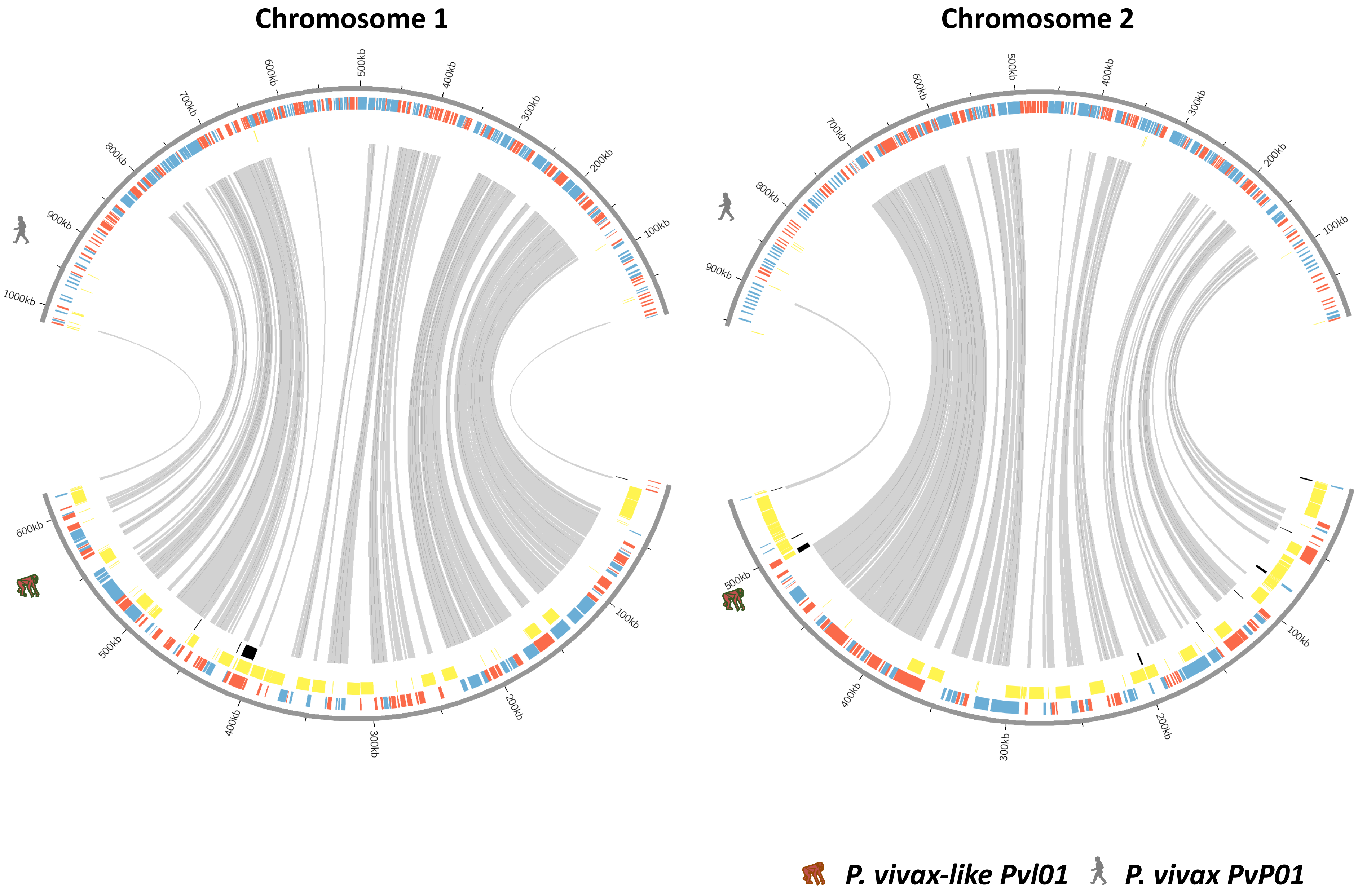

Chromosome 1
Chromosome 2
100kb
200kb
300kb
400kb
500kb
600kb
700kb
800kb
900kb
1000kb
P. vivax-like Pvl01
P. vivax PvP01

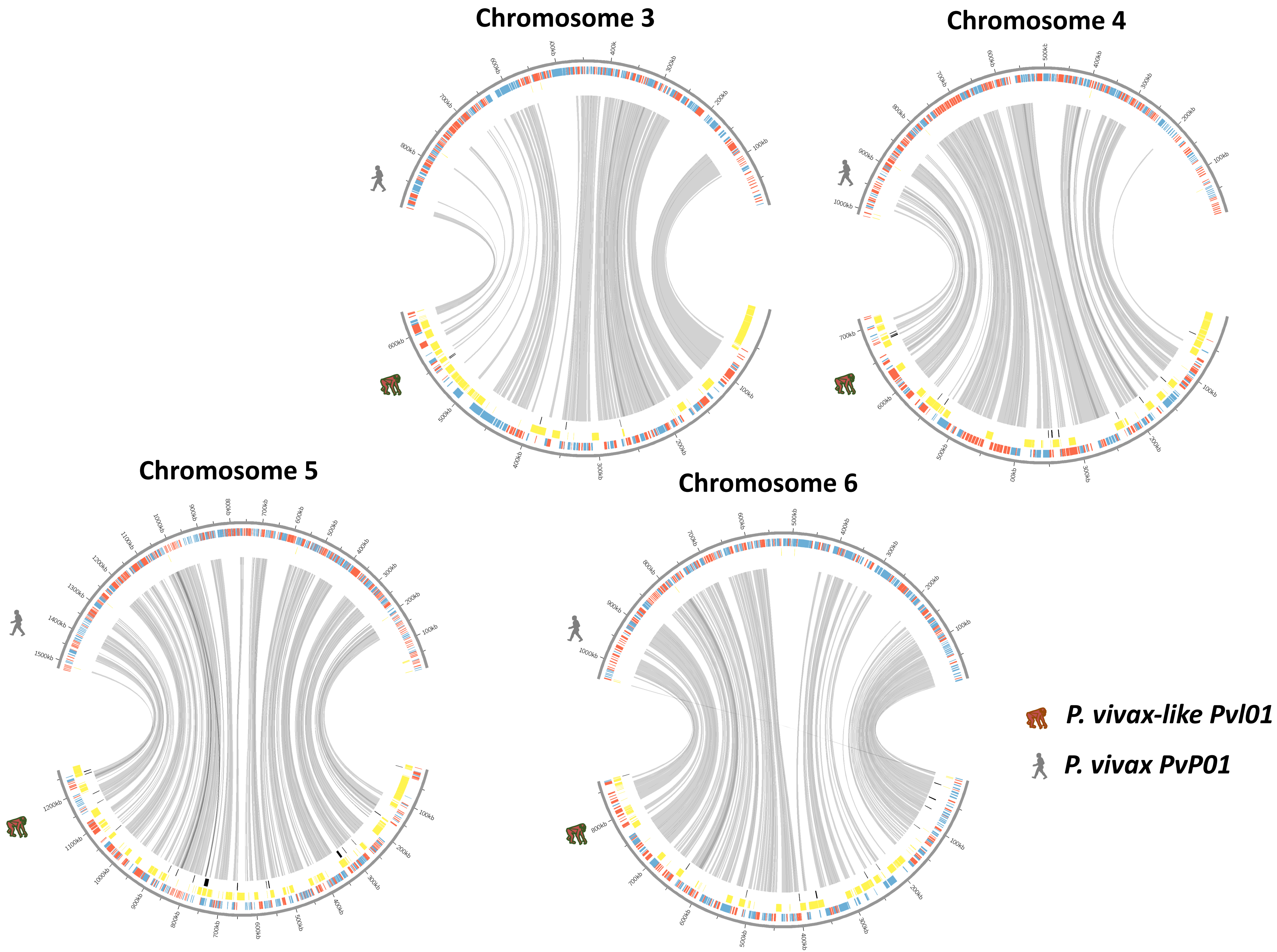

Chromosome 3
Chromosome 4
Chromosome 5
Chromosome 6
P. vivax-like Pvl01
P. vivax PvP01

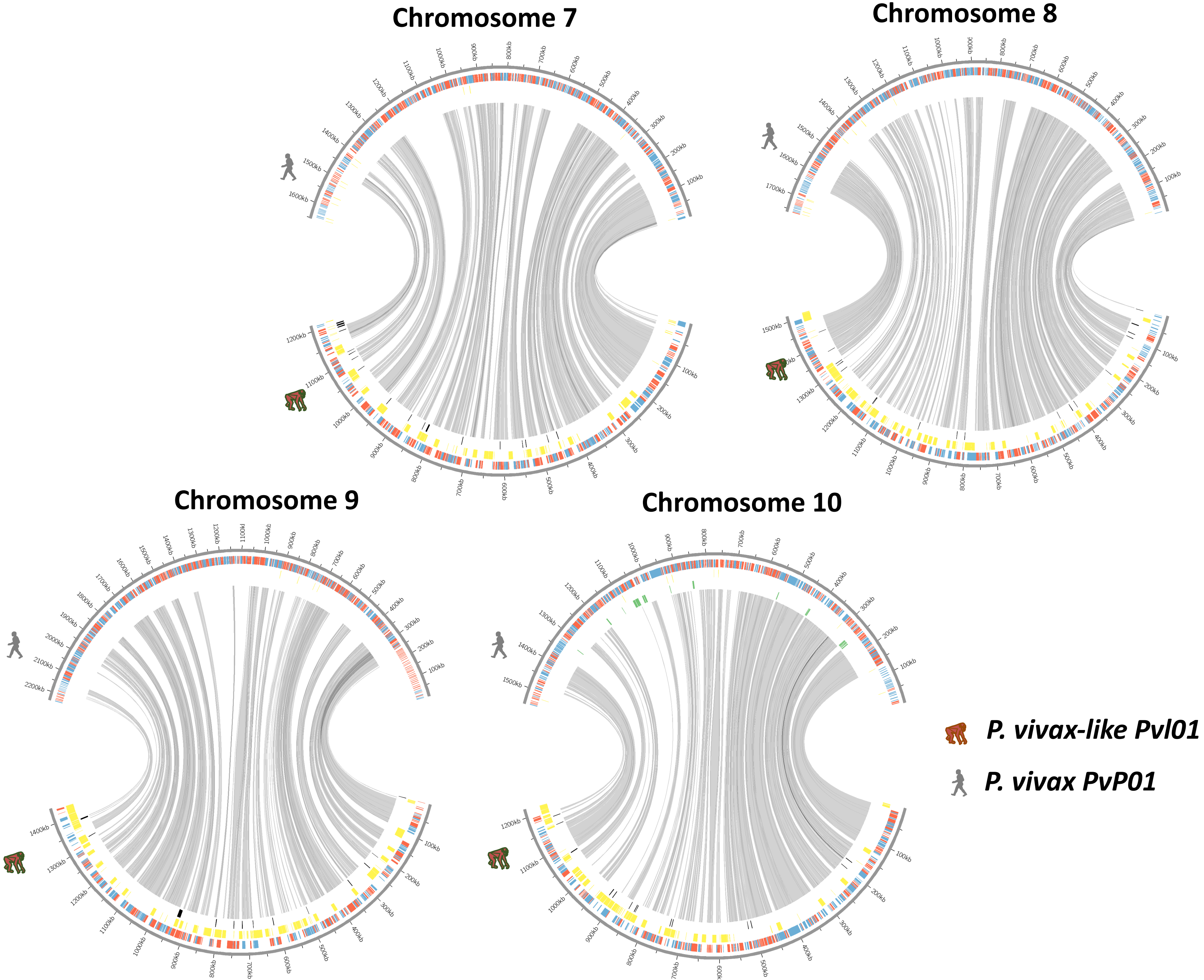
Chromosome 7
Chromosome 8
Chromosome 9
Chromosome 10
P. vivax-like Pvl01
P. vivax PvP01

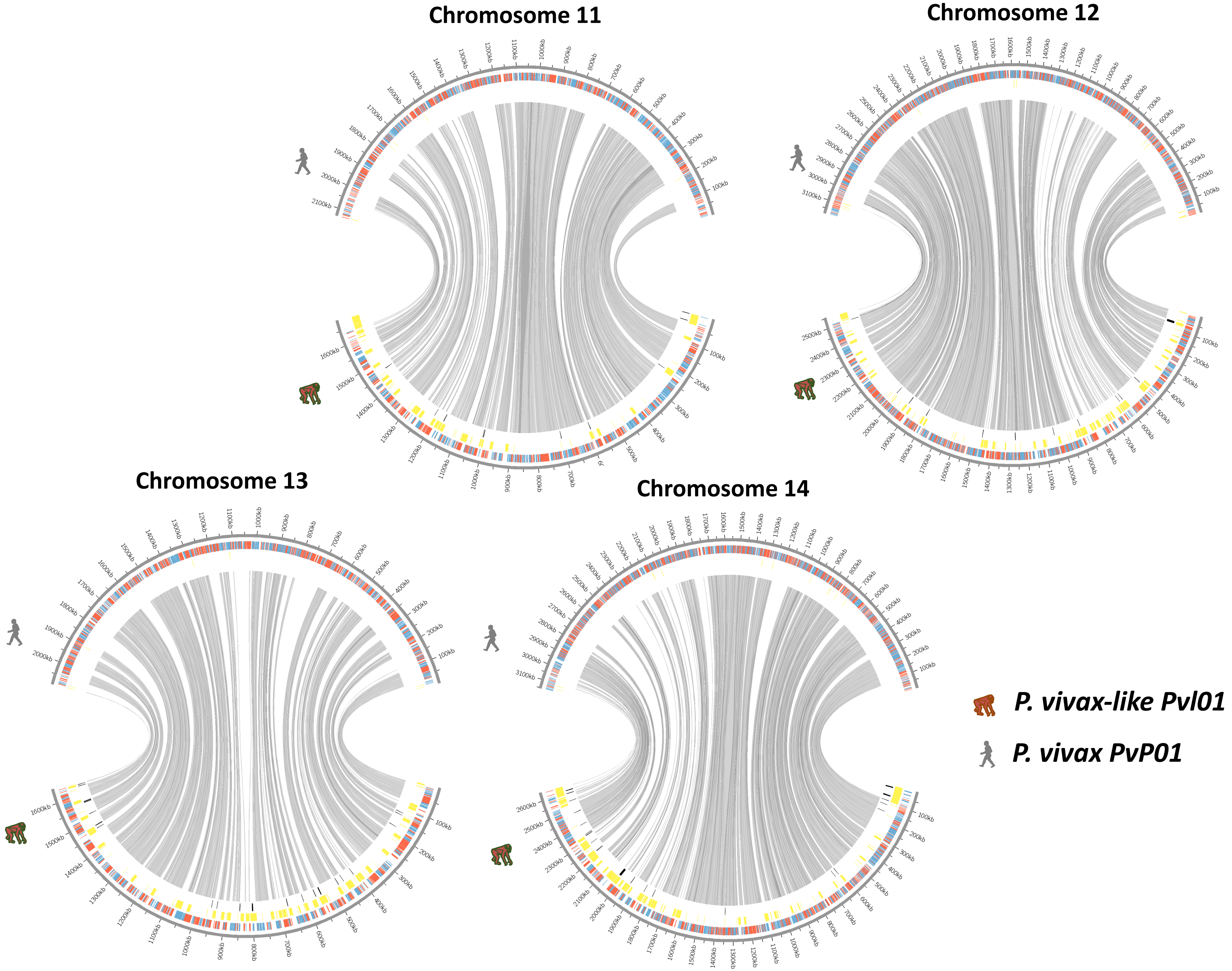
Chromosome 11
Chromosome 12
Chromosome 13
Chromosome 14
P. vivax-like Pvl01
P. vivax PvP01
